# Sex and Gender in Research on Healthcare Workers in Conflict Settings: A Scoping Review

**DOI:** 10.3390/ijerph17124331

**Published:** 2020-06-17

**Authors:** Rima R. Habib, Dana A. Halwani, Diana Mikati, Layal Hneiny

**Affiliations:** 1 Department of Environmental Health, Faculty of Health Sciences, American University of Beirut, Beirut 1107 2020, Lebanon; dah30@mail.aub.edu; 2 Faculty of Medicine, American University of Beirut, Beirut 1107 2020, Lebanon; dm29@aub.edu.lb; 3 Saab Medical Library, American University of Beirut, Beirut 1107 2020, Lebanon; lh32@aub.edu.lb

**Keywords:** healthcare workers, scoping review, war, conflict, sex, gender, occupational health and safety

## Abstract

The occupational health literature has established that sex and gender are associated with all dimensions of the workplace. Sex and/or gender (sex/gender) factors play an important role in shaping the experiences, exposures, and health outcomes of male and female healthcare providers working in war and conflict settings. This study aims to (1) assess how sex/gender is considered in the occupational health literature on healthcare workers in conflict settings, and (2) identify the gaps in incorporating sex/gender concepts in this literature. A scoping review was carried out and nine electronic databases were searched using a comprehensive search strategy. Two reviewers screened the titles/abstracts and full-texts of the studies using specific inclusion and exclusion criteria. Key information was extracted from the studies and four themes were identified. Of 7679 identified records, 47 were included for final review. The findings underlined the harsh working conditions of healthcare workers practicing in conflict zones and showed sex/gender similarities and differences in experiences, exposures and health outcomes. This review revealed a dearth of articles with adequate consideration of sex/gender in the study design. Sex/gender-sensitive research in occupational health is necessary to develop effective occupational health and safety policies to protect men and women healthcare workers in conflict settings.

## 1. Introduction

In 2017, more than 50 armed conflicts were documented in the world, of which, most were concentrated in the Middle East and North Africa (MENA) region including Syria, Iraq, and the Occupied Palestinian Territories, where conflicts have been continuous and chronic [1]. Wars have detrimental impacts on all sectors of life, including social, economic, and environmental. Wars and conflicts have led to the destruction of properties, the displacement of people, but most significantly, the loss of human life [2,3]. Deaths resulting from armed conflicts have increased in the last decade, rising by 118.0% from 2007 to 2017, estimating a total of 130,000 deaths [4].

Healthcare facilities and healthcare professionals are prone to face hazardous and precarious conditions during time of conflict and war [5,6,7]. The loss of medical staff is a primary concern in war settings, resulting from deaths, injuries, or staff leaving the profession [6,7]. During the Syrian crisis, 70% of health workers fled the country seeking safety elsewhere, due to the continuous violent attacks on medical facilities and healthcare workers (HCWs) [8]. The loss of qualified staff forces health facilities to rely on junior and inexperienced staff and volunteers [9]. In fact, most HCWs practicing in conflict settings are not emergency doctors and do not have experience in emergency medicine [6,7]. In conflict settings, HCWs practice with insufficient equipment, materials, and drugs, and lack of basic amenities such as water, electricity, and sanitation [9]. Such is the case with surgical doctors in Yemen, Afghanistan, Syria, and Gaza, who often perform surgeries without anesthetic drugs because of constant shortages in medications and supplies [6,7]. In addition, conflicts and wars often lead to limited access to adequate training, which in turn compromises the medical capabilities and skills of healthcare staff [10].

During times of conflict and war, healthcare facilities become targets of violent attacks; high rates of kidnapping and murder among healthcare workers are reported [7,8]. In 2018, 973 attacks on healthcare workers and healthcare facilities were documented in 23 countries in conflict, with a high incidence of attacks and deaths in the Occupied Palestinian Territories, Syria, Afghanistan, and Yemen. These attacks resulted in 167 deaths and 710 injuries among HCWs, in addition to 173 destroyed healthcare facilities [11]. In Syria, Medecins Sans Frontieres (MSF) reported 373 attacks on 265 medical facilities and the death of 750 medical personnel since the beginning of the conflict in Syria in 2011 until 2016 [12].

Emergency Medical Service (EMS) workers who respond to attacks and disasters also face hazardous working conditions in times of conflict [5]. EMS workers are susceptible to exposures to harmful chemicals, such as rubble and dust from the destruction or collapse of buildings and other structures; these exposures have been associated with physical health problems such as respiratory diseases and disturbances in lung function [13]. Tasks performed by EMS workers, such as lifting, carrying, or transferring patients and/or equipment have also been associated with a number of injuries and health conditions such as musculoskeletal pain, sprains and strains, back pains, and body injuries [13,14]. Moreover, due to the disconcerting and disturbing scenes and exposures EMS workers often encounter during rescue missions, studies have also found higher rates of constant fatigue, low sleep quality, and mental health problems among this workforce [15,16].

Experiences and exposures during wars and conflict often differ between men and women; these differences are attributed to both biological and societal variations for each sex and gender [17]. Sex is a term used to refer to the biological and physiological differences between men and women, while gender is used to refer to the societal roles and expectations of men and women [18]. In wars for instance, men are more likely than women to engage in warrior roles because of cultural norms that attribute the characteristics of toughness and aggression mainly to males [17]. On the other hand, under these norms, women take on the role of “family caregiver” during wars, with the additional burden of assuming the role of “economic provider” [17]. Because of these varying gendered roles, men are at a higher risk of death and torture in combat, while women report higher rates of sexual and domestic violence [17]. Additionally, as studies have shown, women are disproportionately affected by displacement, whereby they are forced to flee their countries along with their children [17].

Similarly, sex and gender play an important role in occupational health. In the workplace, women and men can have different work exposures and health outcomes because of differences in job assignments and work tasks [19,20,21]. The gendered segregation of jobs and tasks are often influenced by societal roles, stereotypes, and expectations [22,23]. This division of labor has led to the emergence of the “feminized” and “masculinized” activities and tasks, materializing in different working conditions and uneven distribution of workplace exposures between men and women workers [24]. On the other hand, studies have shown that male and female workers may respond differently to similar exposures and working conditions because of varying biological, psychological, and environmental vulnerabilities [21,25]. However, the distinction between sex and gender in occupational health research is often blurred, as it can be difficult to identify whether the biological determinants or the social influences are responsible for the observed differences between men and women, particularly in studying job and task assignments [19,26]. For example, when studying the prevalence of mental health problems such as post-traumatic stress disorder (PTSD) among HCWs in conflict settings, should we use the term sex or gender? Do we know how much of the observed difference in disease prevalence between men and women comes from biological determinants (such as genetic predisposition, or chromosomal and chemical variations that may increase the risk of developing mental health conditions) as opposed to social contributors (such as men being viewed as tougher and therefore are more likely to be sent to conflict zones with high exposures to violence or combat)? For this reason, the term “sex/gender” has been used to account for this conflation in research that aims to explore such phenomena; this allows for a more encompassing approach that avoids any assumption that would otherwise be implied by simply selecting either term “sex” or “gender” [26,27]. Similarly, we used the term “sex/gender” to refer to sex and/or gender in this review.

Studies have explored the sex/gender differences among HCWs practicing in different countries, in relation to recruitment, positions, and wages [28]. Estimates show that women predominate the workforce in the health and social work labor markets; a study conducted in 104 countries found that women constitute 70% of the health workforce [29]. Yet, the same study shows varying job and task allocations by gender: males are often in pharmacy, medicine, and dentistry positions, while females are in nursing and midwifery jobs [29]. In recent years, reports show an increasing trend of women engaging in higher level healthcare positions; yet, they still face discrimination and wage disparities of up to 30% compared to their male counterparts [29]. Studies have found that female HCWs have higher rates of work-related musculoskeletal pain [30], and they experience more workplace bullying than their male counterparts [31].

Due to sex/gender differences, the consideration of sex/gender in occupational health research is essential to inform sound evidence-based health and safety policies. Previous reviews have been conducted to examine the occupational health literature on the working conditions and the health outcomes of HCWs in conflict settings [7,8]. To the best of our knowledge, no reviews have been carried out to study how sex/gender considerations are addressed in this literature. To this end, we aimed to (1) assess how sex/gender is considered in the occupational health literature on healthcare workers in conflict settings, and (2) identify the gaps in incorporating sex/gender concepts in this literature.

## 2. Materials and Methods

### 2.1. Study Design

To address our objectives, we conducted a scoping review to map the available literature on the topic and identify the gaps in the published research. Scoping reviews are emerging evidence-mapping tools that allow examination of research output to explore available literature and highlight areas of gaps for analysis [32,33]. We followed the Joanna Briggs Institute guidelines for scoping reviews [34].

### 2.2. Protocol

We developed a protocol for this scoping review following the PRISMA extension for Scoping Reviews (PRISMA-ScR) [35].

### 2.3. Eligibility Criteria

We aimed to reduce the restrictions in the inclusion criteria to explore most of the available literature on healthcare workers in conflict settings. We considered as eligible for this scoping review the following:

▪ Study Design: primary studies including quantitative (e.g., surveys, cross-sectional, cohort, case-control) and qualitative (e.g., interviews, focus groups). We excluded editorials, commentaries, reviews, and studies published only in abstract form.
▪ Population of interest: Healthcare workers
▪ Setting of interest: Conflict setting

A study that did not meet all three criteria was excluded. In addition, articles that were not published in the English language were excluded.

### 2.4. Literature Search

We developed a search strategy with the assistance of a medical librarian (LH, a co-author on this article) and searched 9 electronic databases for our review: Medline, Web of Science, Scopus, EMBASE, Cochrane, PubMed, CINHAL, Global Index Medicus, and Global Health. During the search, no restrictions were applied on years or type of study to capture most articles that have addressed the topic in the literature. The search was conducted on September 18, 2019. We used an inclusive set of keywords, Medical Subject Headings, and Boolean terms, such as “AND” and “OR”, to prepare a comprehensive search strategy. The search strategies are detailed in Supplementary File 1. Four concepts were used in our search: (1) healthcare workers, (2) armed conflict or war setting, (3) sex/gender, and (4) occupational health:

▪ Keywords used for healthcare workers included: doctors, nurses, midwives, occupational therapists, hospital assistants and secretaries, emergency medical personnel, or any type of occupation that is related to healthcare and healthcare settings.
▪ Keywords used for armed conflict included: war, warfare, terrorism, bioterrorism, battle, combat, or any other term used to describe political problems and conflicts causing political instability.
▪ Keywords used for sex/gender included: sex, gender, men, women, male, female, sex/gender factors, sex/gender characteristics, sex/gender similarities, sex/gender differences, and related terms.
▪ Keywords used for occupational health included: occupational health and safety, work safety, industrial hygiene, quality of working life, and other occupational terms used to describe health and safety of workers.

### 2.5. Selection Process

Two stages for the screening and selection of articles were carried out by two reviewers: first the title and abstract screening, and second, the full-text screening. During title and abstract screening, the reviewers used the eligibility criteria to screen separately and independently the records (articles) for potential eligibility. Full-texts were then obtained for all records identified as relevant during the title and abstract screening, and then screened by two reviewers, independently and in duplicate. Discrepancies between the reviewers in the title and abstract screening and the full-text screening were resolved by discussion between the reviewers until a consensus was reached or by consulting a third reviewer. Record screening and management was conducted using the reference management software EndNote X8 (Clarivate Analytics, Philadelphia, USA). Duplicates were removed using the Endnote feature manually. The screening results were reported and documented using Preferred Reporting Items for Systematic Reviews and Meta-Analyses (PRISMA) chart.

### 2.6. Data Abstraction

Data were extracted from the included articles and collected into a data charting form by one of the reviewers. This form was developed and reviewed by the authors. Information relating to study characteristics and results were extracted from each selected study: (1) authors, (2) year of publication (3) study location, (4) study design, (5) study population, (6) sample size, (7) exposure and outcome assessed, and (8) key findings.

### 2.7. Data Synthesis

The research focus in each selected study was identified. The articles were grouped and the key findings were presented by theme in a narrative summary. The general characteristics of the included studies were descriptively analyzed. Our analysis also focused on the integration of sex/gender in the reviewed studies. A protocol was developed to guide the process of identifying the way sex/gender was addressed in the different sections of the articles, as follows:

1. Study sample: The male to female ratio was extracted for each study.
2. Terminology: The terms used to refer to sex/gender of participants were identified along with the definition or justification for the use of the term, if available.
3. Objectives: Studies were assessed on whether the objectives stated the aim to compare men and women or explore sex/gender similarities and differences.
4. Data analysis: Studies were assessed to identify if and how sex/gender was incorporated in the analysis. The categories included: (1) sex/gender was a covariate or confounder in the analysis or (2) the analysis was stratified by sex/gender, in which the exposures and outcomes of interest were analyzed separately for male and female participants.
5. Key findings: Sex/gender-related results were identified where available.
6. Interpretation of findings: Sex/gender-related interpretation of results were identified where available.

## 3. Results

**Figure 1:**
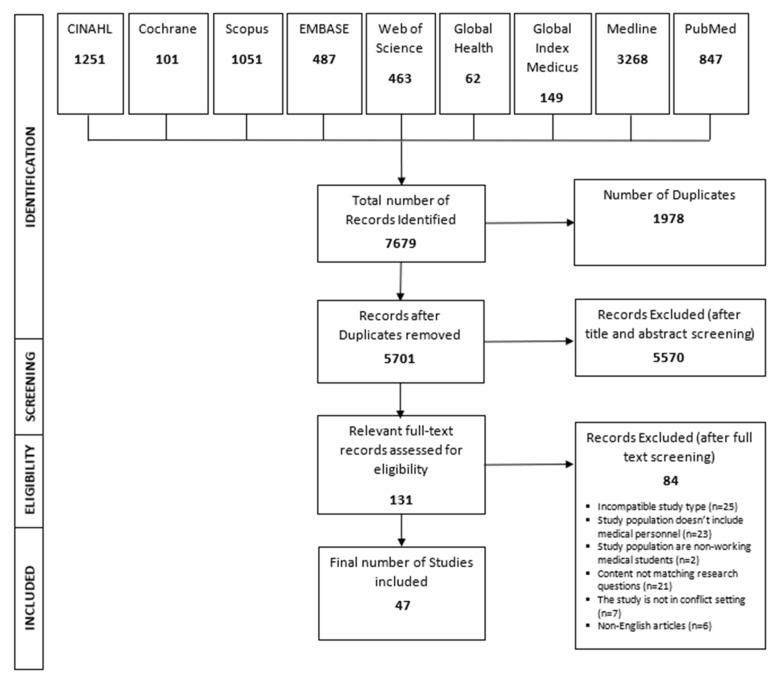
The PRISMA flow diagram presenting the selection process of the scoping review.

The selection process of articles is presented in Figure 1 using the PRISMA flow diagram. Of a total of 7679 records resulting from the search of nine electronic databases, 131 were found to be relevant after the title and abstract screening. A total of 47 articles were included in the analysis after full-text screening (See Supplementary File 2 for a full list of the included studies). The reasons for excluding articles in the full-text screening are listed in Figure 1.

### 3.1. Characterstics of the Studies

**Figure 2:**
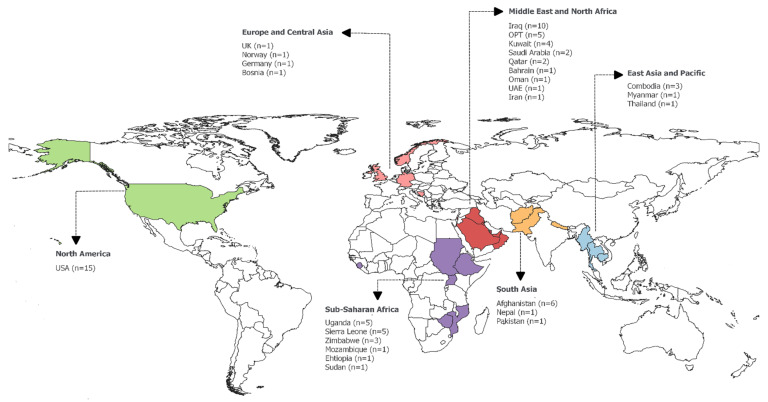
Geographical distribution of the included studies. “n” denotes the number of publications in each country; OPT: Occupied Palestinian Territories; UAE: United Arab Emirates; UK: United Kingdom; USA: United States of America.

Table 1 presents the descriptive characteristics of the studies in this review. The majority of studies were quantitative in nature: 16 (34%) were cross-sectional surveys, 14 (30%) were cohort studies, and two (4%) were case-control. In addition, 12 (26%) studies were qualitative with a cross-sectional design, employing in-depth interviews, focus groups, or participant observations. Only three (6%) studies employed mixed methods. The majority of articles studied healthcare workers in the Middle East and North Africa region (MENA) (n = 15, 32%), mainly Iraq and Palestine, and the North America region (n = 15, 32%), specifically in the United States of America. Nine (19%) and eight (17%) studies were carried out in Sub-Saharan Africa and South Asia, respectively. The remaining articles were distributed among the East Asia and Pacific region (n = 5, 11%) and Europe and Central Asia (n = 3, 6%); and of those, eight articles covered multiple geographical locations (Figure 2 presents a map of the geographical distribution of the studies by country). Healthcare workers in hospital settings were studied in 22 (47%) articles, whereas 16 (34%) articles studied EMS personnel, and nine (19%) focused on deployed military medical staff. In addition, 12 (26%) articles had a sample size of less than 50 participants, three (6%) articles had a sample size between 50 and 99 participants, 18 (38%) articles between 100 and 499, six (13%) between 500 and 999, and 10 (21%) had 1000 or more participants.

Table 2 presents findings on the integration of sex/gender in the included articles. Most studies (n = 39) recruited both male and female participants; 21 studies recruited more males with ratios ranging from 1.47:1 to 6.2:1, 10 studies recruited more females with ratios ranging between 1:1.3 and 1:5.13, and eight had almost equal ratios of males and females ranging between 1:1.05 and 1.2:1. Most studies (n = 7) with almost equal ratios of male and female participants did not justify their sample recruitment strategy; only one study clarified that the researchers aimed to recruit one male and one female participant from each study location (provinces and villages). Of those with unequal ratios, 25 articles did not justify the reason for recruiting unequal number of male and female participants, while only six studies justified the profile of their study sample. The explanations provided were as follows: (1) the male/female ratio of the participants reflects the sex/gender distribution in the workforce, (2) the study inclusion criteria (such as years of service) influenced the male/female ratio of participants, although the study initially aimed to recruit an equal number of male and female participants, and (3) purposive and snowball sampling techniques influenced the male to female ratio in the study sample. Additionally, five articles recruited only male participants and did not provide a justification for this strategy; two others did not specify the sex/gender of the participants; and one article specified the sex/gender of its participants for only a segment of the study sample.

The terminology used to refer to sex/gender of participants varied among the selected studies (Table 2). The majority of the articles (n = 41) did not justify the use of terms: 26 of those articles used the term “gender” without a justification (for example: the social dimension of the term was not alluded to), six articles used the term “sex” without a justification (for example, the biological dimension of the term was not alluded to), and nine articles used both terms interchangeably without explaining the reasons for it. On the other hand, only one study defined the use of the term “gender” as being “multidimensional” encompassing societal, institutional, and personal aspects. Additionally, five articles did not use the terms “sex” and “gender”; of those, two articles used the terms “men” and “women” to refer to the study participants, and three articles used the terms “male” and “female” (of which one included the terms “male” and “female” in the keywords only, and not in the text of the article) [10].

Our review also assessed how sex/gender was incorporated in the objectives, analysis, results, and discussion of the research articles (Table 2). Our findings showed eight studies that had specific objectives relating to sex/gender exploration. In addition, from a total of 32 quantitative studies, 19 incorporated sex/gender in the analysis: 12 of those had sex/gender as a confounder or a covariate in multivariate models. Four studies used sex/gender as an explanatory variable in univariate models, and three articles stratified their analysis by sex/gender. In addition, four studies did not consider sex/gender in statistical models, and four had only descriptive analyses. Of a total of 15 qualitative and mixed methods studies, only three analyzed sex/gender differences. In addition, 30 studies reported on sex/gender-specific outcomes; of those, only 15 interpreted their sex/gender-related results.

### 3.2. Main Themes Covered in the Studies

Four main themes or topics of study were identified in the reviewed articles: (1) working conditions including job allocations and roles, as well as the experiences and challenges faced by HCWs practicing in conflict settings (n = 21); (2) workplace violence (n = 3); (3) physical health (n = 15); and (4) mental and social health (n = 23). Table 3 presents the covered themes and key findings.

#### 3.2.1. Working Conditions

Working conditions of healthcare workers in conflict settings were assessed in 21 (45%) studies. Among the articles that discussed sex/gender as a factor in shaping the work experiences of HCWs, eight studies found that females predominate low-paying positions, including nursing, midwifery and community health workers (CHWs), as well as other unpaid positions, while male workers predominate managerial and policy-maker positions [36,37,38,39,40,41,42,43]. These studies attributed the difference in the allocation of job positions to gendered power relations, norms, and stereotypes, such that females are more likely to take on CHW jobs due to limited job opportunities and societal biases that see women only in care-giving roles [39]. Furthermore, studies reported that females are underrepresented in managerial positions due to lower educational levels attained, and difficulties in accessing professional training and development, as well as additional hindrances in promotion that males do not face [36,37,41]. Despite these differences, two studies found that both male and female HCWs commonly reported the escape from poverty, higher salaries, personal calling, status, and a sense of deservingness as motivations for joining the profession [39,40].

Six studies reported that both male and female HCWs face similar difficulties when working in conflict zones, which include limited supplies and equipment, insufficient medications, shortage of qualified personnel, and increase in workload and working days, as well as low or lack of pay, and economic insecurity [41,42,44,45,46,47]. Studies also showed similar exposures to dangerous conditions and physical hazards, including threats, harassment, injury, death due to combat exposure, attacks on healthcare facilities, as well as arrests and direct assault for both male and female HCWs [44,46,48,49,50,51,52]. Other exposures, such as chemical hazards including dust and smoke containing air pollutants, toxic clouds, particulate matter, and demolition rubble and dust [53,54,55,56,57], as well as psychological hazards including trauma [38,58], were studied. These studies did not report differences in exposures between male and female HCWs.

Only one study aimed to identify sex/gender differences in the perceived work stressors among deployed HCWs; factors that increased stress were common among both male and female participants, which included fear of fire, terrorist attacks, dying, death of colleagues, and fear of the unknown [59]. However, increased stress levels were reported by females compared to males; this study attributed these sex/gender differences to unmeasured variables in men and women [59]. Three additional studies found that separation from family for long periods of time was demotivating for female workers in conflict settings, especially in isolated rural areas with poor communication [40,43,48]. Other demotivating factors were common for both male and female HCWs, including limited financial incentives, difficult working conditions, and lack of career progression [43,48]. A study exploring the experiences of HCWs under the governance of the Islamic State of Iraq and Syria (ISIS) found that males and females were strictly separated with limited communication and restriction of movement, particularly for females due to the implementation of strict religious norms [60].

#### 3.2.2. Workplace Violence

Three (6%) articles focused on workplace violence, of which one studied sex/gender differences in physical and non-physical workplace violence among medical staff, and reported the lack of sex/gender differences [61]. This study reported higher non-physical than physical violence (for example verbal abuse, threats, and sexual harassment) among both male and female HCWs [61]. The other two articles found that males reported higher levels of exposure to physical violence (27.6% of males vs. 16.2% of female [62], and 75% of males vs. 24.2% of females [63]). These studies attributed the differences to social and cultural norms that prevent discussing and reporting physical violence against women in the study region [62,63].

#### 3.2.3. Physical Health

Physical illnesses were studied in 15 (32%) articles. Only in one article did the study objectives state the aim to compare health outcomes between males and females; the findings showed no differences in lung function and recovery among male and female participants [64]. Another study reported the prevalence of rhinosinusitis, gastroesophageal reflux disease, and obstructive airway disease among emergency medical staff, with increased rates reported among females compared to males (for example, incidence of rhinosinusitis was 17% for females compared to 8.9% for males) [65]. Male and female emergency medical staff with exposure to violence and terrorism reported a number of health problems and poor health [63,66], but males were found to report poorer health compared to females in a scale used to measure perceived general health (62.4% in males vs. 66.7% in females) [66].

Almost all of the other studies pooled the results for both males and females for specific health outcomes and found physical health problems in EMS and other HCWs. The reported health outcomes included chronic respiratory illnesses, allergies, asthma, gastroesophageal problems, eye problems, neurological problems, musculoskeletal and systematic auto-immune disorders, and cancer [51,54,55,56,67,68,69,70,71]. In addition, a study found decreased lung functions in male and female EMS workers [53].

#### 3.2.4. Mental and Social Health

Twenty-three articles (49%) focused on mental and social health. Two studies aimed to examine sex/gender differences in the prevalence of post-traumatic stress disorder (PTSD) among deployed HCWs; both reported higher rates of PTSD among females compared to males (35.3% in females vs. 30.8% in males [72], 6.6% in females vs. 3% in males [38]). Interpretation of these sex/gender-related findings attributed the differences to biological, psychosocial, and societal variations, military etiologies, and varying sex/gender roles within the same occupation [38,72]. Another study similarly reported increased levels of PTSD and depression in female workers, although it had not initially intended to explore sex/gender differences among HCWs (PTSD rates: 10.4% in females vs. 6.1% in males, and depression rates: 23.5% in females vs. 14.8% in males) [65]. Similarly, females were found to be at a higher risk for post-deployment mental health (PDMH) conditions compared to males [73].

In contrast, other studies showed no sex/gender differences for the incidence of PTSD and depression, and no significant sex/gender associations for other psychological outcomes such as probable autism spectrum disorder (ASD), depression, stress and other PDMH conditions [55,74,75,76,77,78]. However, one of these studies reported differences in mental health help-seeking behaviors with a higher proportion of females (100%) seeking help for PTSD than males (36%) [55]. In addition, one study attributed the lack of the sex/gender differences to the fact that the PDMH conditions were self-reported and not diagnosed medically, and additionally found that being a nurse and a medical technician was associated with increased reporting of PDMH conditions, as compared to other health professions [76]. Two studies that did not report on sex/gender differences for specific health outcomes found that PTSD, distress, anxiety, and depression were reported by both male and female workers [58,60]. Trauma, distress, and mental health problems were reported by both male and female workers to affect their overall wellbeing [79].

Sleep disturbance was found to be the most common symptom of PTSD [72]. The study reporting this result also found that after return from deployment, women had higher reporting of conflict in their relationships, specifically higher rates of separation and divorce, compared to men (19.1% of females vs. 14.7% of males) [72]. Increased levels of burnout with regards to emotional exhaustion, depersonalization, and reduced personal accomplishment were reported among male and female HCWs [80,81], with higher prevalence in males (12.7%) compared to females (7.9%) [81].

Three studies that had only male participants revealed that probable PTSD, depression, and anxiety were prevalent among male emergency medical responders exposed to terrorist attacks [69,71,82]. Development of mental health problems was also reported by male and female HCWs as a direct consequence of exposure to violence while practicing in conflict zones [63]. One study found a significant association between having a role in a disaster scene and increased reporting of PTSD among EMS workers [75]. Similarly, another study reported that exposure to a disaster or terrorist attack was associated with decreased social functioning and mental health [66]. The same study showed that perceived mental health status was poorer for males than females, attributing this finding to higher prevalence of injury and witnessing deaths [66]. Reported PTSD and depression, in both male and female workers, were associated with decreased mental health measured by the health-related quality of life (HrQOL) scale [71].

A number of articles studied the coping strategies and stress reduction methods adopted by HCWs in conflict settings with few differences detected between males and females. Four studies found that individual resources (such as optimism, spirituality, religiosity, hope, resilience, commitment, and trust in personal abilities), psychological support, sharing with friends and co-workers, borrowing money, and working in two jobs to cope with low wages and payment disruptions, were all coping strategies reported equally by both male and female HCWs [46,48,66,79]. On the other hand, another study that reported stress reducing factors among genders found that men reported writing mail, and women preferred going to the library, as strategies to reduce stress, although both resorted to reading [59].

## 4. Discussion

This scoping review identified the experiences and health burdens among men and women healthcare workers practicing in conflict zones. A large proportion of the reviewed research was in the Middle East and North Africa (MENA) region, where a number of countries have suffered from chronic armed conflicts; examples are Iraq, Palestine, and, more recently, Syria [8]. The majority of articles adopted a cross-sectional design in studying the working conditions of HCWs in conflict settings.

### 4.1. Working Conditions

Our findings show that male and female healthcare workers practicing in conflict settings experience hardship, fear, and precarious working conditions. They are the subject of attacks, kidnapping, and torture. They also face challenges in healthcare delivery, including the lack of equipment and essential medical supplies, poor infrastructure, and shortage of staff [7,8]. Studies have postulated that the weaponization of health care, defined as using health care as a weapon of war by denying it to people in need, has led to increased rates of attacks on healthcare facilities and the imprisonment, torture, and murder of HCWs [7]. As a result, HCWs are forced to migrate to other countries, leaving the nation’s health sector compromised and the health services diminished. For example, violence against HCWs has led to critical deficiencies in medical care in the Democratic Republic of Congo, claiming the lives of 40,000 people per month [83]. In Somalia on the other hand, estimates have shown that a single attack on doctors in 2009 eliminated 150,000 annual physician consultations [83]. For this reason, the International Committee of the Red Cross (ICRC) has proposed pre-negotiations between conflicting armed forces and governments in conflict settings to provide safer working conditions for HCWs on the ground [5].

Many studies in our review reported that females predominate the healthcare profession in conflict zones [36,37,39,40]. These results are consistent with general findings in the health sector, where the World Health Organization estimated that women account for 70% of workers in the healthcare and social sector [29]. In addition, most of the studies that explored the gendered workforce found variations in job allocation among males and females. Women were more likely to work in low-paying and low-status occupations, mainly related to maternal and child health services and nursing, while men were more likely to work in high-wage jobs, such as doctors, and occupy managerial and decision-making positions [36,37,39,40,84].

The majority of the reviewed studies did not find major sex/gender differences in assessing work exposures (work hazards, risks, stressors) among male and female healthcare workers, with only one study reporting higher stress levels in females compared to males. Yet, numerous occupational health studies have proven that work exposures, risks, and health outcomes vary between males and females due to biological and physiological differences, as well as context-specific societal norms [23]. Thus, it is possible that the studies in this scoping review were deficient in employing strategies that were conducive to detecting discrepancies among male and female HCWs, and thus could not tease out the specificities of each sex/gender.

### 4.2. Workplace Violence

Workplace violence in healthcare workers has been extensively studied in the occupational health literature [85,86,87]. Healthcare workers in conflict settings reported both physical and non-physical workplace violence. Concerns over healthcare workers’ physical and mental health resulting from exposure to violence inflicted by their co-workers, patients, and visitors have been reported in the literature [85,86]. Violence to HCWs is likely to exacerbate in the contexts of wars and conflicts due to intense stress and aggressive environments. This has been reflected in the reviewed studies that reported on HCWs’ exposure to both physical and non-physical violence such as direct attacks and threats [61,62,63].

While studies in this review found that physical violence was more common among male HCWs practicing in conflict zones [62,63], another study found no sex/gender differences in exposure to violence [61]. However, findings in the occupational health literature on healthcare workers in various workplace settings showed that males reported higher exposures to physical violence and abuse [86,87] and females reported higher exposures to non-physical violence, such as verbal abuse, threats, and sexual harassment [14,87]. A study on close-to-community health service providers revealed that insecurity was a prevalent and recurring theme for female workers, due to high risks of gender-based violence and sexual harassment in certain communities, which become more prominent in conflict-ridden areas [88]. This highlights how male and female experiences of violence and threats can differ by context and environment, which warrants further research to capture the full reality.

### 4.3. Physical Health

Neurological and respiratory problems, gastroesophageal disorders, and musculoskeletal disorders have been reported among male and female healthcare workers, specifically EMS workers involved in disaster scenes [55,64,65,71]. While one study reported no sex/gender differences in physical health conditions, other studies reported women having higher prevalence of illnesses than men. These contradictions may suggest that each finding is specific to the context in which the study was conducted, and it highlights that sex/gender differences in occupational health studies are often complex and multidimensional, making the generalization of results challenging. It is worth noting that studies that assessed health outcomes in emergency, rescue, and recovery workers by pooling the results and without stratifying their analysis by type of occupation, did not identify the specific tasks performed by males and females in each occupation. As a result, they were limited in identifying the underlying reasons for the differences between male and female workers.

### 4.4. Mental and Social Health

The mental and social health of HCWs was a main focus in the reviewed literature. A high prevalence of mental health disorders, mainly PTSD, stress, and depression, was reported in male and female healthcare workers practicing in conflict settings. This was expected given the hardships and challenging working conditions endured by HCWs in these settings.

However, our results highlight inconsistencies among articles reporting on sex/gender differences in mental health outcomes, such as PTSD, depression, anxiety, and other PDMH conditions. For instance, four studies found higher prevalence rates of stress and mental health disorders, such as PTSD and depression, among female workers compared to males [38,65,72,73], while six studies did not find sex/gender differences [55,74,75,76,77,78]. Meanwhile, evidence suggests that women are at a higher risk of mental health disorders, both generally and during war [3,89]. These gendered differences may be attributed to women acquiring more responsibilities during war, such as becoming income providers while their partners are away fighting in battles, which adds burden to their role as caregivers and makes them more prone to mental health problems [3]. Other contributing factors could be the possible variations in socioeconomic status between male and female healthcare workers in high and low wage positions [89]. In addition, mental health help-seeking behavior was more common in females than males. This may be due to the embedded socially constructed gender roles, pervasive perceptions of mental health, and the different behavioral responses to dealing with personal struggles and mental health conditions [90]. On the other hand, the majority of studies did not find sex/gender differences in assessing coping strategies among male and female HCWs. In general, the reviewed studies did not delve deeper to explore the possible causes or explanations for differences or similarities in mental health conditions and related behaviors in male and female healthcare workers.

### 4.5. Integration of Sex/Gender in the Included Studies

In this scoping review, we identified variations in the way sex/gender was conceptualized and incorporated in the published research. Some studies, though only a few, included only male participants in their research, and others did not specify the sex/gender of their participants, thus overlooking the possibility of exploring relevant sex/gender-related differences in their study. More than half of the reviewed studies had higher proportions of male participants than females, with some articles having six times more men than women, without providing justification for the disparate ratios. Only a few studies clarified that the proportions of male and female participants reflected the actual sex/gender distribution of the study population. The variations in male to female ratios across articles reflect an insufficient effort to recruit participants from both genders.

With regards to terminologies used, almost all articles used either “sex” or “gender” or both interchangeably without providing a definition of the biological and social dimensions of the terms or a justification for their use. This reflects the lack of sex/gender considerations in the formulation of research questions and study design.

While the majority of articles recruited both male and female participants in their study, only a few had well-defined objectives related to exploring sex/gender similarities or differences. Based on their objectives, some articles that did not aim to analyze sex/gender factors ultimately reported sex/gender-related results. The variation in the results on men and women may have influenced the researchers to segregate their findings. Only a few studies interpreted their sex/gender-related results to explain the underlying causes of these differences.

Although few studies stratified their analyses by sex/gender, a sizeable proportion of the studies pooled men and women in the analysis accounting for sex/gender as a confounder or using it as a covariate. This finding reflects a methodological limitation compromising the ability of research in identifying distinctions between men and women HCWs in terms of exposures and outcomes. In studying risk factors for musculoskeletal disorders among the Canadian working population, Messing et al. (2009) employed both sex/gender stratified and un-stratified statistical models [91]. The authors found that a number of risk factors could be missed, overlooked, or overestimated in the un-stratified model that pooled men and women in the analysis [91].

In addition, almost all of the studies did not distinguish between the tasks performed by male and female healthcare workers in conflict zones. This limits the ability of research to identify the underlying causes for the differences in work-related health outcomes between men and women. Detailed exposure assessments unravel the sex/gender differences in workplace exposures. For instance, a study of hospital employees with the same job title showed that men had more physically demanding tasks, while women had more repetitive tasks, which ultimately resulted in different health outcomes [21,92]. In addition, wars and armed conflicts may have distinct burdens on men and women; studying these differences in relation to job type, task distribution, exposures, and health outcomes in men and women HCWs is essential, yet lacking in most of the articles in this review.

### 4.6. Implication for Future Research

Over the past two decades, the occupational health literature has emphasized the importance of integrating sex/gender factors in designing and implementing occupational health research [93,94,95]. In fact, integrating sex/gender in all of the phases of research, starting with conceptualization, statement of the hypothesis and objectives, selection of the study design, recruitment of participants, data collection and analysis, and interpretation of the results, has provided researchers a way to understand the interplay between exposures and outcomes [26]. Accordingly, this has paved the road to planning effective interventions on the health and safety of workers [23,95].

While much of the research today is incorporating sex/gender factors in occupational health studies, many studies continue to ignore it, as evidenced by the publications identified in this review. Conducting appropriate sex/gender-sensitive research and adopting sex and gender-sensitive analyses is vital in developing effective occupational health and safety interventions that may require different control measures for both women and men [21]. This approach entails capturing sex/gender differences in jobs and tasks, and their relationships to biological and physiological variations, and to societal roles and expectations [22,84,96]. To accomplish this, researchers ought to collect accurate sex/gender-related data to inform evidenced-based occupational health and safety policies [22,96]. The most important aspect of sex/gender-sensitive research is attempting to explain how biological and social differences between men and women interact and impact workers’ health [22].

More recent studies have challenged the notion that sex/gender in occupational health is an independent variable or indicator that can be used separately to assess exposures or risk [94]. Instead, these studies argue that sex or gender considerations are context-specific, and they are part of an interconnected dynamic web of structural and social determinants of health. Heise et al. (2019), present a conceptual framework for the gender system in health, which comprises five major pillars with direct and indirect interactions: (1) sex and biological determinants, (2) the gender system and the social production of gender (community context, political and legal frameworks, family influence), (3) gendered social positioning due to age, race, ethnicity, class, and ability, (4) different gendered pathways to health, such as exposures, behaviors, access to healthcare, and research, and (5) health inequities and outcomes [97]. Accordingly, researchers propose that an intersectional approach to sex/gender analysis in occupational health would be critical to explore the intersections of all the individual, social, and contextual characteristics that may impact health outcomes for workers [88,94,98]. This would be particularly crucial in studying occupational health outcomes of HCWs in conflict settings, where the conditions of war and conflict have a major impact on the living and occupational environment and the health of workers. In addition, given that ongoing conflicts in areas such as Palestine, Iraq, Syria, and Yemen have been prolonged for a long duration of time, an intersectional approach would allow a better understanding of the various nuances of socio-cultural determinants and gender norms within the changing historical context [88].

### 4.7. Strengths and Limitations of the Study

This review has three key strengths. First, and to the best of our knowledge, this is the first review conducted to explore and map how sex/gender is integrated in this literature. In addition, this review was carried out following the PRISMA-ScR protocol format to ensure the utilization of standardized and rigorous methods of review. Finally, we searched multiple major databases with no restriction on year of publication to expand our search and capture most relevant articles.

Similar to other scoping reviews, this study also has methodological limitations; it did not critically appraise the quality of evidence and perform in-depth analyses of the included studies; rather it provided a narrative description of the available literature. However, this method was appropriate to meet our objectives in exploring, mapping, and identifying the gaps in the available literature on this topic. Another potential limitation of this review is including only articles that were published in the English language. Finally, the diversity of the study populations, measurement and assessment tools, and outcomes assessed hindered our ability to sufficiently compare findings from different studies.

## 5. Conclusions

The findings of this scoping review underlined the harsh working conditions of HCWs practicing in conflict zones, and explored the sex/gender differences in physical and mental health conditions, workplace violence, and working conditions between male and female healthcare workers. While some of the identified studies incorporated sex/gender factors in their research on exposures and outcomes among HCWs in conflict zones, others continue to ignore sex and gender factors, generalizing the findings to both men and women, or by including only male participants, neglecting to report the sex/gender of the participants, or failing to report sex/gender-related findings. Moreover, among the studies that reported sex/gender-related findings, many did not have clear objectives to capture sex/gender differences or similarities, nor did they interpret sex/gender-related findings. Therefore, our review reveals a gap in the literature, identifying a lack of articles focused on exploring and understanding sex/gender differences. It also highlighted the limited use of sex/gender-sensitive analytical methods in occupational health studies on HCWs in conflict settings, to assist in revealing hidden sex/gender differences that are often overlooked. Sex/gender-sensitive research in occupational health is necessary to ensure the development and implementation of effective occupational health and safety policies that protect healthcare workers in conflict zones and reduce gender inequalities among these front liners assisting communities in their battle of survival.

## Figures and Tables

**Table 1:** Descriptive characteristics of the included articles (n = 47).

Characteristics	Number of Studies	Percentage
Study design		
Quantitative studies		
Cross-sectional surveys	16	34
Cohort	14	30
Case-control	2	4
Qualitative studies		
Cross-sectional	12	26
Mixed methods	3	6
Region *		
Middle East and North Africa	15	32
North America	15	32
Sub-Saharan Africa	9	19
South Asia	8	17
East Asia and Pacific	5	11
Europe and Central Asia	3	6
Type of healthcare workers		
Healthcare staff in hospital settings	22	47
Emergency medical service personnel	16	34
Deployed military medical staff	9	19
Sample size *		
<50	12	26
50–99	3	6
100–499	18	38
500–999	6	13
≥1000	10	21
Theme *		
Mental and social health	23	49
Working conditions	21	45
Physical health	15	32
Workplace violence	3	6

* Percentages do not add up to 100% as categories are not mutually exclusive.

**Table 2:** Integration of sex/gender in the included articles (n = 47).

Sex/Gender Considerations	Number of Studies	Percentage
Male/female ratio in study participants *		
More males than females ^1^	21	43
More females than males ^2^	10	23
Almost equal ^3^	8	17
Male only ^4^	5	11
Sex/gender not specified	3	6
Justification for male/female ratio in sample		
Almost equal		
Yes	1	2
No	7	15
Unequal ratios		
Yes	6	13
No	25	53
Only male participants		
Yes	0	0
No	5	6
Not Applicable **	3	6
Term used to refer to sex/gender of participants		
Sex	6	14
Gender	27	57
Sex and gender	9	19
Male/female	3	6
Men/women	2	4
Justification for the use of sex and gender terms		
Sex		
Yes	0	0
No	6	13
Gender		
Yes	1	2
No	26	55
Sex and gender		
Yes	0	0
No	9	19
Not Applicable ***	5	11
Sex/gender-related objectives		
Yes	8	17
No	39	83
Sex/gender in the analysis		
Quantitative Analyses		
Confounder or covariate in multivariate models	12	26
Explanatory variable in univariate models	4	9
Stratified analysis by sex/gender	3	6
No consideration of sex/gender in statistical models	4	9
No statistical models—only descriptive analysis	4	9
Males only in the study	5	11
Qualitative Analyses		
Sex/gender analysis	3	6
No sex/gender analysis	12	26
Sex/gender-related findings		
Yes	30	64
No	17	36
Interpretation of sex/gender-related findings ****		
Yes	1	32
No	15	32

* Percentages do not add up to 100% as categories are not mutually exclusive; ^1^ M/F ratio range 1.47:1–6.2:1; ^2^ M/F ratio range 1:1.31–1:5.13; ^3^ M/F ratio range 1:1.05–1.2:1; ^4^ M/F ratio range 1:0; ** Sex/gender not specified in the articles; *** Articles did not use the terms sex or gender, they used male/female or men/women; **** Only for the 30 articles that reported results by sex/gender.

**Table 3:** Sex/gender-related key findings of included articles by theme.

Theme	Sex/Gender-Related Key Findings
Working Conditions	Females predominate low-paying and un-paid healthcare positions; males predominate managerial and policy-maker positions Motivations for joining the profession include: escape from poverty, higher salaries, personal calling, status, and a sense of deservingness for both males and females Work challenges include: limited supplies and equipment, insufficient medications, shortage of qualified personnel, increase in workload, and economic insecurity for both males and females Exposure to environmental and occupational hazards including: (1) physical hazards including violence, injury and combat exposures, (2) chemical hazards including dust and smoke containing air pollutants, toxic clouds, particulate matter, and demolition rubbles, and (3) psychological hazards including trauma for both males and females Stress-related exposures for both males and females—increased levels reported by females Demotivating factors for practicing in conflict zones including limited financial incentives, difficult working conditions, and lack of career progression for both males and females; separation from family more demotivating for females
Workplace Violence	Exposure to workplace violence for both males and females; males reported more exposure to physical violence
Physical Health	Poorer health status reported by males compared to females Health conditions including: chronic respiratory illnesses, allergies, asthma, gastroesophageal problems, eye problems, neurological problems, musculoskeletal and systematic auto-immune disorders, and cancer reported by both males and females Rhinosinusitis, gastroesophageal reflux disease, obstructive airway disease reported more among females than males Lung function decline for both male and female emergency medical workers
Mental and Social Health	Post-traumatic stress disorder (PTSD), anxiety, depression, and probable autism spectrum disorder reported by both male and female deployed HCWs; increased rated of PTSD, depression, and post-deployment mental health in females compared to males Increased levels of burnout in males compared to females Increased rates of separation and divorce after return from deployment for females compared to males Mental health help-seeking behavior more predominant in females than in males (example: seeking help for PTSD) Coping strategies and stress reduction methods including: individual resources (such as optimism, spirituality, religiosity, hope, resilience, commitment, and trust in personal abilities), psychological support, sharing with friends and co-workers, borrowing money, and working in two jobs, for both males and females

## Supplementary Materials

The following are available online at https://www.mdpi.com/1660-4601/17/12/4331/s1, Supplementary File 1: Search Strategies, Supplementary File 2: List if included Studies.
