# Supplementary material for: Sex and Gender in Research on Healthcare Workers in Conflict Settings: A Scoping Review
Source: Int J Environ Res Public Health. 2020 Jun 17;17(12):4331. doi: 10.3390/ijerph17124331 (PMC7346087; doi:10.3390/ijerph17124331)
Supplement: Supplementary file 1 [file ijerph-17-04331-s001.pdf]

Supplementary File 1: Search Strategies

Date of Searches: 18 September 2019

Database #1: The Cumulative Index to Nursing and Allied Health Literature (CINAHL) Complete –1937 to September 2019

(MH "War+") OR (MH "TerrORism+") OR (MH "Weapons+") OR (MH "TORture") OR (MH "Refugees") OR (MH "Transients and Migrants") OR TI (battlefield# OR ((conflict# OR battle# OR combat#) N3 (field# OR environment# OR setting# OR context# OR zone#)) OR conflict* OR (fragile N2 state*) OR terrORism OR bioterrORis* OR genocide# OR (ethnic* W1 (violence* OR cleans*))) OR AB (battlefield# OR ((conflict# OR battle# OR combat#) N3 (field# OR environment# OR setting# OR context# OR zone#)) OR conflict* OR (fragile N2 state*) OR terrORism OR bioterrORis* OR genocide# OR (ethnic* W1 (violence* OR cleans*))) OR TI (violence* OR holocaust# OR Gun# OR Rifle# OR shotgun# OR firearm# OR weapon# OR bomb# OR explosive# OR bullet* OR (humanitarian N2 cris#s) OR (complex W1 (emergenc* OR (security W1 environment*))) OR refugee# OR ((displaced OR undocumented OR un-documented OR transient OR vulnerable) N2 (population# OR group# OR nation# OR people OR person*)) OR (asylum W1 seeker#)) OR AB (violence* OR holocaust# OR Gun# OR Rifle# OR shotgun# OR firearm# OR weapon# OR bomb# OR explosive# OR bullet* OR (humanitarian N2 cris#s) OR (complex W1 (emergenc* OR (security W1 environment*))) OR refugee# OR ((displaced OR undocumented OR un-documented OR transient OR vulnerable) N2 (population# OR group# OR nation# OR people OR person*)) OR (asylum W1 seeker#)) OR TI ((fORced W1 displacement) OR migrant* OR postconflict* OR post-conflict* OR postwar* OR post-war* OR ((conflict OR war) W1 affected) OR immigrant* OR emigrant* OR ((warfare OR war# OR ((fORced OR armed) W1 conflict#)) N2 (victim# OR survivOR#))) OR AB ((fORced W1 displacement) OR migrant* OR postconflict* OR post-conflict* OR postwar* OR post-war* OR ((conflict OR war) W1 affected) OR immigrant* OR emigrant* OR ((warfare OR war# OR ((fORced OR armed) W1 conflict#)) N2 (victim# OR survivOR#))) OR TI ((militar* W1 attack#) OR tORture OR Warfare# OR war OR wars OR ((operation* W1 ((new W1 dawn#) OR ((Iraq* OR endur*) W1 freedom) OR (desert W1 (shield OR stORm)))) OR (afghan* W1 campaign W1 "2001") OR ((French OR American) N2 revolution*) OR (9-11-2001 W1 attack#) OR ((September OR Sep) N3 attack#))) OR AB ((militar* W1 attack#) OR tORture OR Warfare# OR war OR wars OR ((operation* W1 ((new W1 dawn#) OR ((Iraq* OR endur*) W1 freedom) OR (desert W1 (shield OR stORm)))) OR (afghan* W1 campaign W1 "2001") OR ((French OR American) N2 revolution*) OR (9-11-2001 W1 attack#) OR ((September OR Sep) N3 attack#))) AND (MH "Sexism") OR (MH "Gender-Based Violence") OR (MH "Gender Identity+") OR (MH "Sex FactORs") OR (MH "Men") OR (MH "Male") OR (MH "Female") OR (MH "Women") OR TI ((“gender based violence” OR “gender-based violence” OR sexism OR (discriminat* OR injust* OR intoleran* OR insensitiv* OR harass* OR bias* OR favo#riti#m# OR budget* OR mainstream* OR stereotyp* OR issue OR favORing OR unfairness* OR in-equit* OR inequit* OR unequal OR un-equal)) N4 (wom#n OR m#n OR female# OR male# OR gender# OR sex)) OR AB ((“gender based violence” OR “gender-based violence” OR sexism OR (discriminat* OR injust* OR intoleran* OR insensitiv* OR harass* OR bias* OR favo#riti#m# OR budget* OR mainstream* OR stereotyp* OR issue OR favORing OR unfairness* OR in-equit* OR inequit* OR unequal OR un-equal)) N4 (wom#n OR m#n OR female# OR male# OR gender# OR sex)) OR TI ((sex* N2 factOR*) OR (sexual W1 dimORphism*) OR masculin* OR feminin* OR ((characteristic* OR differen* OR d#mORphism# OR similarit* OR compar* OR outcome* OR exposure* OR perspective* OR perception* OR perceive* OR sensitive* OR dynamic* OR risk factOR* OR stratification* OR segregation* OR conflict* OR variab* OR diversit* OR relation* OR variation* OR approach* OR disparit* OR analys#s* OR role*) N3

(gender# OR sex OR (male# OR m#n OR (female# OR wom#n))))) OR AB ((sex* N2 factOR*) OR (sexual W1 dimORphism*) OR masculin* OR feminin* OR ((characteristic* OR differen* OR d#mORphism# OR similarit* OR compar* OR outcome* OR exposure* OR perspective* OR perception* OR perceive* OR sensitive* OR dynamic* OR risk factOR* OR stratification* OR segregation* OR conflict* OR variab* OR diversit* OR relation* OR variation* OR approach* OR disparit* OR analys#s* OR role*) N3 (gender# OR sex OR (male# OR m#n OR (female# OR wom#n))))) AND (MH "Health Personnel+") OR (MH "Students, Health Occupations+") OR TI (physician* OR doctOR* OR allopath# OR osteopath# OR homeopath# OR ((clinical* OR health* OR medical) W1 (practitioner# OR specialist# OR generalist#)) OR ((medical OR nurs* OR dental) W1 (student# OR graduate# OR intern# OR fellow#)) OR (house W1 officer#) OR Resident# OR nurse# OR nursing OR ORderly OR ORderlies OR dieti#ian* OR psychologist* OR counselOR* OR therapist* OR pharmacist* OR dentist* OR hygeinist* OR phlebotomist* OR chiropracter* OR acupuncturist* OR an#esthesiologist* OR an#esthetist* OR perfusionist* OR surgeon* OR p#ediatrician* OR obstetrician* OR gynaecologist* OR gynecologist* OR radiologist* OR sonographer* OR cardiologist* OR gastroenterologist* OR hepatologist* OR endocrinologist* OR diabetologist* OR internist* OR hospitalist* OR intensivist* OR rheumatologist* OR immunologist* OR dermatologist* OR allergist* OR otolaryngologist* OR otORhinolaryngologist* OR oncologist* OR h#ematologist* OR ORthop#edist* OR physiatrist* OR podiatrist* OR pathologist* OR diener* OR cytologist* OR cytogeneticist* OR geneticist* OR h#ematopathologist* OR neurologist* OR neurosurgeon* OR ophthalmologist* OR optician* OR urologist* OR nephrologist* OR pulmonologist* OR neonatologist* OR psychiatrist* OR physiotherapist* OR dentist* OR prosthodontist* OR periodontist* OR endodontist OR nutritionist* OR optometrist* OR geriatrician* OR gerontologist* OR otologist* OR feldsher* OR exodontist* OR doula* OR (labOR N3 coach*) OR MD OR anatomist* OR audiologist* OR cORoner#) OR AB (physician* OR doctOR* OR allopath# OR osteopath# OR homeopath# OR ((clinical* OR health* OR medical) W1 (practitioner# OR specialist# OR generalist#)) OR ((medical OR nurs* OR dental) W1 (student# OR graduate# OR intern# OR fellow#)) OR (house W1 officer#) OR Resident# OR nurse# OR nursing OR ORderly OR ORderlies OR dieti#ian* OR psychologist* OR counselOR* OR therapist* OR pharmacist* OR dentist* OR hygeinist* OR phlebotomist* OR chiropracter* OR acupuncturist* OR an#esthesiologist* OR an#esthetist* OR perfusionist* OR surgeon* OR p#ediatrician* OR obstetrician* OR gyn#ecologist* OR radiologist* OR sonographer* OR cardiologist* OR gastroenterologist* OR hepatologist* OR endocrinologist* OR diabetologist* OR internist* OR hospitalist* OR intensivist* OR rheumatologist* OR immunologist* OR dermatologist* OR allergist* OR otolaryngologist* OR otORhinolaryngologist* OR oncologist* OR h#ematologist* OR ORthop#edist* OR physiatrist* OR podiatrist* OR pathologist* OR diener* OR cytologist* OR cytogeneticist* OR geneticist* OR h#ematopathologist* OR neurologist* OR neurosurgeon* OR ophthalmologist* OR optician* OR urologist* OR nephrologist* OR pulmonologist* OR neonatologist* OR psychiatrist* OR physiotherapist* OR dentist* OR prosthodontist* OR periodontist* OR endodontist OR nutritionist* OR optometrist* OR geriatrician* OR gerontologist* OR otologist* OR feldsher* OR exodontist* OR doula* OR (labOR N3 coach*) OR MD OR anatomist* OR audiologist* OR cORoner#) OR TI (((health* OR hospital OR medical OR clinic* OR inpatient* OR outpatient*) N3 (examiner* OR assistant# OR administratOR* OR supervisOR* OR receptionist* OR personnel OR team OR staff OR wORker* OR researcher OR researchers OR librarian* OR infORmationist* OR technician* OR manager* OR housekeeper* OR janitOR* OR officer* OR responder* OR paramedic* OR labORatORian* OR coORdinatOR* OR navigatOR* OR attendant* OR scientist* OR cashier# OR accountant# OR registrar# OR provider* OR chaperone# OR man-power OR manpower OR wORkfORce* OR (care W1 giver*) OR caregiver#)) OR epidemiologist* OR ((ER OR emergency OR "911") W1 dispatcher*) OR midwi#e#) OR AB (((health* OR hospital OR medical OR clinic* OR inpatient* OR outpatient*) N3 (examiner* OR assistant# OR administratOR* OR supervisOR* OR receptionist* OR personnel OR team OR staff OR wORker*

OR researcher OR researchers OR librarian* OR infORmationist* OR technician* OR manager* OR housekeeper* OR janitOR* OR officer* OR responder* OR paramedic* OR labORatORian* OR coORdinatOR* OR navigatOR* OR attendant* OR scientist* OR cashier# OR accountant# OR registrar# OR provider* OR chaperone# OR man-power OR manpower OR wORkfORce* OR (care W1 giver*) OR caregiver#)) OR epidemiologist* OR ((ER OR emergency OR "911") W1 dispatcher*) OR midwi#e#) AND (MH "Occupational Health") OR TI ((wORker# OR employee# OR occupational OR industrial) N3 (hazard* OR exposure* OR health OR safety OR hygiene)) OR AB ((wORker# OR employee# OR occupational OR industrial) N3 (hazard* OR exposure* OR health OR safety OR hygiene)) OR TI ((aged N2 wORker*) OR employabilit* OR (wORk* N2 (capacit* OR abilit* OR resumption* OR schedule* OR table* OR time* OR timing# OR week* OR day# OR load)) OR wORkload OR wORk-load OR wORkday# OR wORk-day# OR wORkweek# OR wORk-week* OR 'quality of wORking life') OR AB ((aged N2 wORker*) OR employabilit* OR (wORk* N2 (capacit* OR abilit* OR resumption* OR schedule* OR table* OR time* OR timing# OR week* OR day# OR load)) OR wORkload OR wORk-load OR wORkday# OR wORk-day# OR wORkweek# OR wORk-week* OR 'quality of wORking life')

Database #2: Cochrane Library – 1992 to September 2019

(MeSH descriptor [Torture] this term only OR MeSH descriptor [Terrorism] explode all trees OR MeSH descriptor [Warfare and Armed Conflicts] explode all trees OR MeSH descriptor [Explosive Agents] explode all trees OR MeSH descriptor [Weapons] explode all trees OR MeSH descriptor [Transients and Migrants] this term only OR MeSH descriptor [Refugees] this term only OR MeSH descriptor [Human Migration] explode all trees) OR (Battlefield* OR ((conflict* OR battle* OR combat*) NEAR/3 (field* OR environment OR environments OR setting* OR context OR contexts OR zone*)) OR conflict* OR (fragile NEAR/2 state*) OR terrorism* OR bioterrorism* OR genocide* OR (ethnic NEAR/1 (violence OR cleans*)) OR violence OR holocaust* OR gun* OR rifle* OR shotgun* OR firearm* OR weapon* OR bomb* OR explosive* OR bullet* OR (humanitarian NEAR/2 (crisis OR crises)) OR ((displaced OR undocumented OR transient OR vulnerable) NEAR/2 (population* OR group* OR nation* OR people OR person*)) OR migrant* OR postconflict* OR post-conflict* OR postwar* OR post-war* OR conflict-affected OR war-affected OR immigrant* OR emigrant* OR warfare* OR war* OR (armed NEXT/1 conflict*) OR (militar* NEAR/1 attack*) OR torture OR ((Iraq OR endur*) NEXT/1 freedom) OR (desert NEXT/1 (shield OR storm)) OR (afghan NEXT/1 campaign NEXT/1 “2001”) OR ((French OR American) NEAR/2 revolution) OR (“9 11 2001” NEXT/1 attack*) OR ((September OR sep) NEAR/3 attack*) OR (complex NEXT/1 emergenc*) OR (complex NEXT1 security NEXT/1 environment) OR refugee OR (asylum NEXT/1 seeker*) OR (forced NEXT/1 displacement*)):ti,ab,kw AND (MeSH descriptor [Sexism] this term only OR MeSH descriptor [Gender Identity] explode all trees OR MeSH descriptor [Sex Factors] this term only OR MeSH descriptor [Sex Characteristics] this term only OR MeSH descriptor [Men] this term only OR MeSH descriptor [Women] this term only) OR (sexism OR ((characteristic* OR differen* OR dimorphism* OR dimorphism* OR similarit* OR compar* OR outcome* OR exposure* OR perspective* OR perception* OR perceive* OR sensitive* OR dynamic* OR stratification* OR segregation* OR conflict* OR variable* OR diversit* OR relation* OR variation* OR approach* OR disparit* OR analysis OR analyses OR role*) NEAR/3 (gender OR sex OR male OR Males OR m?n OR female OR females OR wom?n)) OR ((discriminat* OR injust* OR intoleran* OR insensitive* OR harass* OR bias* OR favo?ritism* OR budget* OR mainstream* OR stereotyp* OR issue* OR favoring OR unfairness OR in-equit* OR inequit* OR un-equal* OR unequal*) NEAR/4 (wom?n OR m?n OR female OR male OR gender OR sex)) OR (sex NEAR/2 factor) OR masculin* OR feminin* OR (gender NEXT/1 risk NEXT/1 factor*) OR (sex NEXT/1 risk NEXT/1 factor*) OR (m?n NEXT/1 risk NEXT/1 factor*) OR (male NEXT/1 risk NEXT/1 factor*) OR (female NEXT/1 risk NEXT/1 factor*) OR (wom?n NEXT/1 risk NEXT/1 factor*) OR (sexual NEXT/1 dimorphism*)):ti,ab,kw AND (MeSH descriptor [Occupational Health]

this term only) OR (‘quality of working life’ OR (work* NEAR/2 (capacit* OR abilit* OR resumption OR schedule* OR table* OR time* OR timing OR timings OR week* OR day OR days OR load)) OR (aged NEAR/2 worker) OR ((worker OR workers OR employee OR employees OR occupational OR industrial) NEAR/3 (hazard* OR exposure* OR health OR safety OR hygiene))):ti,ab,kw AND (MeSH descriptor [Health Personnel] explode all trees OR MeSH descriptor [Students, Health Occupations] explode all trees OR MeSH descriptor [Health Workforce] this term only) OR (((medical OR nurs* OR dental) NEXT/1 (student* OR graduate* OR intern OR Interns OR fellow OR fellows)) OR (labor NEAR/3 coach*) OR md OR anatomist* OR audiologist* OR coroner* OR ((health OR hospital OR medical OR clinic* OR inpatient OR outpatient) NEAR/3 (examiner* OR assistant* OR administrator* OR supervisor* OR receptionist* OR personnel OR team OR staff OR worker* OR researcher* OR researchers OR librarian* OR informationist* OR technician* OR manager* OR housekeeper* OR janitor* OR officer* OR responder* OR paramedic* OR laboratorian* OR coordinator* OR navigator* OR attendant* OR scientist* OR cashier* OR accountant* OR registrar OR registrars OR provider* OR chaperone OR chaperones OR man-power OR manpower OR workforce* OR caregiver*)) OR ((er OR emergency OR “911”) NEXT/1 dispatcher*) OR epidemiologist* OR midwife OR midwives OR (medical NEXT/1 care NEXT/1 giver*) OR (hospital NEXT/1 care NEXT/1 giver*) OR (clinical NEXT/1 care NEXT/1 giver*) OR (house NEXT/1 officer*) OR resident OR residents OR nurse OR nurses OR nursing OR orderly OR orderlies OR dietitian* OR dietician* OR psychologist* OR counselor* OR therapist* OR pharmacist OR hygeinist OR phlebotomist OR chiropracter OR acupuncturist OR anesthesiologist* OR anaesthesiologist* OR anesthetist* OR anaesthetist* OR perfusionist* OR surgeon* OR pediatrician* OR paediatrician* OR obstetrician* OR gynecologist* OR gynaecologist* OR radiologist* OR sonographer* OR cardiologist* OR gastroenterologist* OR hepatologist* OR endocrinologist* OR diabetologist* OR internist* OR hospitalist* OR intensivist* OR rheumatologist* OR immunologist* OR dermatologist* OR allergist* OR otolaryngologist* OR otorhinolaryngologist* OR oncologist* OR hematologist* OR haematologist* OR orthopedist* OR orthopaedist* OR physiatrist* OR podiatrist* OR pathologist* OR diener* OR cytologist* OR cytogeneticist* OR geneticist* OR hematopathologist* OR haematopathologist* OR neurologist* OR neurosurgeon* OR ophthalmologist* OR optician* OR urologist* OR nephrologist* OR pulmonologist* OR neonatologist* OR psychiatrist* OR physiotherapist* OR dentist* OR prosthodontist* OR periodontist* OR endodontist* OR nutritionist* OR optometrist* OR geriatrician* OR gerontologist* OR otologist* OR feldsher* OR exodontist* OR doula*):ti,ab,kw

Database #3: EMBASE – 1947 to September 2019

('torture'/exp OR 'war'/exp OR 'warfare'/exp OR 'terrorism'/exp OR 'explosive'/exp OR 'refugee'/exp OR 'migrant'/exp OR 'migration'/exp) OR battlefield$:ti,ab,kw OR (((conflict$ OR battle$ OR combat$) NEAR/3 (field$ OR environment$ OR setting$ OR context$ OR zone$)):ti,ab,kw) OR conflict*:ti,ab,kw OR ((fragile NEAR/2 state*):ti,ab,kw) OR terrorism:ti,ab,kw OR bioterroris*:ti,ab,kw OR genocide$:ti,ab,kw OR ((ethnic* NEAR/1 (violence* OR cleans*)):ti,ab,kw) OR violence*:ti,ab,kw OR holocaust$:ti,ab,kw OR gun$:ti,ab,kw OR rifle$:ti,ab,kw OR shotgun$:ti,ab,kw OR firearm$:ti,ab,kw OR weapon$:ti,ab,kw OR bomb$:ti,ab,kw OR explosive$:ti,ab,kw OR bullet*:ti,ab,kw OR ((humanitarian NEAR/2 cris$s):ti,ab,kw) OR (((displaced OR undocument* OR transient OR vulnerable) NEAR/2 (population$ OR group$ OR nation$ OR people OR person*)):ti,ab,kw) OR migrant*:ti,ab,kw OR postconflict*:ti,ab,kw OR 'post conflict':ti,ab,kw OR 'post conflicts':ti,ab,kw OR postwar*:ti,ab,kw OR 'post war':ti,ab,kw OR 'post wars':ti,ab,kw OR 'conflict affected':ti,ab,kw OR 'war affected':ti,ab,kw OR immigrant*:ti,ab,kw OR emigrant*:ti,ab,kw OR warfare:ti,ab,kw OR war$:ti,ab,kw OR ((armed NEXT/1 conflict*):ti,ab,kw) OR ((militar* NEAR/1 attack$):ti,ab,kw) OR torture:ti,ab,kw OR warfare$:ti,ab,kw OR war:ti,ab,kw OR wars:ti,ab,kw OR (((iraq* OR

endur*) NEXT/1 freedom):ti,ab,kw) OR ((desert NEXT/1 (shield OR storm)):ti,ab,kw) OR ((afghan* NEXT/1 campaign NEXT/1 '2001'):ti,ab,kw) OR (((french OR american) NEAR/2 revolution*):ti,ab,kw) OR (('9 11 2001' NEXT/1 attack$):ti,ab,kw) OR (((september OR sep) NEAR/3 attack$):ti,ab,kw) OR ((complex NEXT/1 emergenc*):ti,ab,kw) OR ((complex NEXT/1 security NEXT/1 environment*):ti,ab,kw) OR refugee*:ti,ab,kw OR ((asylum NEXT/1 seeker$):ti,ab,kw) OR ((forced NEXT/1 displacement):ti,ab,kw) AND ('sexism'/exp OR 'gender identity'/exp OR 'sex factor'/exp OR 'sexual characteristics'/exp) OR (((characteristic* OR differen* OR d$morphism$ OR similarit* OR compar* OR outcome* OR exposure* OR perspective* OR perception* OR perceive* OR sensitive* OR dynamic* OR stratification* OR segregation* OR conflict OR variabl* OR diversit* OR relation* OR variation* OR approach* OR disparit* OR analys$s OR role*) NEAR/3 (gender$ OR sex OR male$ OR m$n OR female$ OR wom$n)):ti,ab,kw) OR sexism:ti,ab,kw OR (((discriminat* OR injust* OR intoleran* OR insensitiv* OR harass* OR bias* OR favo$riti$m$ OR budget* OR mainstream* OR stereotyp* OR issue OR favoring OR unfairness* OR 'in equity' OR 'inequities' OR inequit* OR unequal OR 'un equal') NEAR/4 (wom$n OR m$n OR female$ OR male$ OR gender$ OR sex)):ti,ab,kw) OR ((sex* NEAR/2 factor*):ti,ab,kw) OR masculin*:ti,ab,kw OR feminin*:ti,ab,kw OR ((gender NEXT/1 risk NEXT/1 factor):ti,ab,kw) OR ((sex NEXT/1 risk NEXT/1 factor):ti,ab,kw) OR ((m$n NEXT/1 risk NEXT/1 factor):ti,ab,kw) OR ((male$ NEXT/1 risk NEXT/1 factor):ti,ab,kw) OR ((female$ NEXT/1 risk NEXT/1 factor):ti,ab,kw) OR ((wom$n NEXT/1 risk NEXT/1 factor):ti,ab,kw) OR ((sexual NEXT/1 dimorphism*):ti,ab,kw) OR 'gender based violence':ti,ab,kw AND ('health care personnel'/exp OR 'health student'/exp) OR (((medical OR nurs* OR dental) NEXT/1 (student$ OR graduate$ OR intern$ OR fellow$)):ti,kw) OR ((labor NEAR/3 coach*):ti,ab,kw) OR md:ti,ab,kw OR anatomist*:ti,ab,kw OR audiologist*:ti,ab,kw OR coroner$:ti,ab,kw OR (((health* OR hospital OR medical OR clinic* OR inpatient* OR outpatient*) NEAR/3 (examiner* OR assistant$ OR administrator* OR supervisor* OR receptionist* OR personnel OR team OR staff OR worker* OR researcher OR researchers OR librarian* OR informationist* OR technician* OR manager* OR housekeeper* OR janitor* OR officer* OR responder* OR paramedic* OR laboratorian* OR coordinator* OR navigator* OR attendant* OR scientist* OR cashier$ OR accountant$ OR registrar$ OR provider* OR chaperone$ OR 'man power' OR manpower OR workforce* OR caregiver$)):ti,ab,kw) OR (((er OR emergency OR '911') NEXT/1 dispatcher*):ti,ab,kw) OR epidemiologist*:ti,ab,kw OR midwi$e*:ti,ab,kw OR ((medical NEXT/1 care NEXT/1 giver$):ti,ab,kw) OR ((hospital NEXT/1 care NEXT/1 giver$):ti,ab,kw) OR ((clinical NEXT/1 care NEXT/1 giver$):ti,ab,kw) OR ((house NEXT/1 officer$):ti,ab,kw) OR resident$:ti,ab,kw OR nurse$:ti,ab,kw OR nursing:ti,ab,kw OR orderly:ti,ab,kw OR orderlies:ti,ab,kw OR dieti$ian*:ti,ab,kw OR psychologist*:ti,ab,kw OR counselor*:ti,ab,kw OR therapist*:ti,ab,kw OR pharmacist*:ti,ab,kw OR hygeinist*:ti,ab,kw OR phlebotomist*:ti,ab,kw OR chiropracter*:ti,ab,kw OR acupuncturist*:ti,ab,kw OR an$esthesiologist*:ti,ab,kw OR an$esthetist*:ti,ab,kw OR perfusionist*:ti,ab,kw OR surgeon*:ti,ab,kw OR p$ediatrician*:ti,ab,kw OR obstetrician*:ti,ab,kw OR gyn$ecologist*:ti,ab,kw OR radiologist*:ti,ab,kw OR sonographer*:ti,ab,kw OR cardiologist*:ti,ab,kw OR gastroenterologist*:ti,ab,kw OR hepatologist*:ti,ab,kw OR endocrinologist*:ti,ab,kw OR diabetologist*:ti,ab,kw OR internist*:ti,ab,kw OR hospitalist*:ti,ab,kw OR intensivist*:ti,ab,kw OR rheumatologist*:ti,ab,kw OR immunologist*:ti,ab,kw OR dermatologist*:ti,ab,kw OR allergist*:ti,ab,kw OR otolaryngologist*:ti,ab,kw OR otorhinolaryngologist*:ti,ab,kw OR oncologist*:ti,ab,kw OR h$ematologist*:ti,ab,kw OR orthop$edist*:ti,ab,kw OR physiatrist*:ti,ab,kw OR podiatrist*:ti,ab,kw OR pathologist*:ti,ab,kw OR diener*:ti,ab,kw OR cytologist*:ti,ab,kw OR cytogeneticist*:ti,ab,kw OR geneticist*:ti,ab,kw OR h$ematopathologist*:ti,ab,kw OR neurologist*:ti,ab,kw OR neurosurgeon*:ti,ab,kw OR ophthalmologist*:ti,ab,kw OR optician*:ti,ab,kw OR urologist*:ti,ab,kw OR nephrologist*:ti,ab,kw OR pulmonologist*:ti,ab,kw OR neonatologist*:ti,ab,kw OR psychiatrist*:ti,ab,kw OR physiotherapist*:ti,ab,kw OR dentist*:ti,ab,kw OR prosthodontist*:ti,ab,kw OR periodontist*:ti,ab,kw OR endodontist:ti,ab,kw OR

nutritionist*:ti,ab,kw OR optometrist*:ti,ab,kw OR geriatrician*:ti,ab,kw OR gerontologist*:ti,ab,kw OR otologist*:ti,ab,kw OR feldsher*:ti,ab,kw OR exodontist*:ti,ab,kw OR doula*:ti,ab,kw AND ('occupational health'/exp) OR 'quality of working life':ti,ab,kw OR ((work* NEAR/2 (capacit* OR abilit* OR resumption* OR schedule* OR table* OR time* OR timing$ OR week* OR day$ OR load)):ti,ab,kw) OR ((aged NEAR/2 worker*):ti,ab,kw) OR (((worker$ OR employee$ OR occupational OR industrial) NEAR/3 (hazard* OR exposure* OR health OR safety OR hygiene)):ti,ab,kw)

Database #4: Global Health – 1998 to September 2019

((("Sex factor" OR "sex factors" OR "sexual dimorphism" OR "sexual dimORphisms" OR masculin OR masculinity OR feminine OR feminin OR femininism OR femininity OR "gender characteristic" OR "gender characteristics" OR "gender difference" OR "gender differences" OR "gender similarity" OR "gender similarities" OR "gender comparison" OR "gender comparisons" OR "gender outcome" OR "gender outcomes" OR "gender exposure" OR "gender exposures" OR "gender perspective" OR "gender perspectives" OR "gender perception" OR "gender perceptions" OR "sex characteristic" OR "sex characteristics" OR "sex difference" OR "Sex differences" OR "sex similarity" OR "sex similarities" OR "sex comparison" OR "sex comparisons" OR "sex outcome" OR "sex outcomes" OR "sex exposure" OR "sex perspective" OR "sex perspectives" OR "sex perception" OR "sex perceptions" OR "men difference" OR "men differences" OR "men similarity" OR "men similarities" OR "men exposure" OR "men exposures" OR "man comparison" OR "man exposure" OR "man exposures" OR "male characteristic" OR "male difference" OR "male similarity" OR "male comparison" OR "male outcome" OR "male exposure" OR "male perspective" OR "male perception" OR "female characteristic" OR "female difference" OR "female comparison" OR "female outcome" OR "female exposure" OR "female perspective" OR "female perception" OR "women characteristic" OR "women difference" OR "women comparison" OR "women outcome" OR "women exposure" OR "women perspective" OR "women perception" OR "woman characteristic" OR "woman difference" OR "woman similarity" OR "woman comparison" OR "woman outcome" OR "woman perception" OR "man comparisons" OR "man outcomes" OR "man exposures" OR "male characteristics" OR "male differences" OR "male similarities" OR "male comparisons" OR "male outcomes" OR "male exposures" OR "male perspectives" OR "male perceptions" OR "female characteristics" OR "female differences" OR "female comparisons" OR "female outcomes" OR "female exposures" OR "female perspectives" OR "female perceptions" OR "women characteristics" OR "women differences" OR "women comparisons" OR "women outcomes" OR "women exposures" OR "women perspectives" OR "women perceptions" OR "woman characteristics" OR "woman differences" OR "woman similarities" OR "woman comparisons" OR "woman outcomes" OR "woman perceptions" OR "Gender sensitive" OR "gender dynamic" OR "gender dynamics" OR "gender risk factor" OR "gender risk factors" OR "gender stratification" OR "gender segregation" OR "gender segregations" OR "gender conflicts" OR "gender conflicts" OR "gender variable" OR "gender variables" OR "gender diversity" OR "gender diverse" OR "gender relation" OR "gender relations" OR "gender variation" OR "Gender variations" OR "gender approach" OR "gender disparity" OR "gender disparities" OR "gender analysis" OR "gender analyses" OR "gender role" OR "gender roles" OR "sex sensitive" OR "sex dynamic" OR "Sex dynamics" OR "sex risk factor" OR "sex risk factors" OR "sex stratification" OR "sex segregation" OR "sex variable" OR "sex variables" OR "sex diversity" OR "sex relation" OR "sex relations" OR "sex variation" OR "sex variations" OR "sex disparity" OR "sex disparities" OR "sex analysis" OR "sex analyses" OR "sex role" OR "sex roles" OR "male sensitive" OR "male sensitives" OR "male risk factor" OR "male risk factors" OR "male diversity" OR "male relation" OR "male relations" OR "male variation" OR "male approach" OR "male approaches" OR "male role" OR "men sensitive" OR "male roles" OR "man sensitive" OR "man roles" OR "man role" OR "female sensitive" OR "female risk factor" OR "female risk factors" OR "female variable" OR

"female variables" OR "female relation" OR "female relations" OR "female variation" OR "female variations" OR "female approach" OR "female analysis" OR "female analyses" OR "female role" OR "female roles" OR "women sensitive" OR "women analysis" OR "women analyses" OR "woman sensitive" OR "woman role" OR "woman roles" OR sexism OR "female harass" OR "female harassment" OR "female stereotype" OR "female stereotyping" OR "female stereotypical" OR "female stereotypes" OR "female stereotypes" OR "female stereotypic" OR "female issue" OR "female issues" OR "female favoring" OR "male discrimination" OR "male discriminations" OR "male discriminate" OR "male harass" OR "male harassment" OR "male bias" OR "male biases" OR "male biased" OR "male biasing" OR "male stereotype" OR "male stereotyping" OR "male stereotypical" OR "male stereotypes" OR "male stereotypes" OR "male stereotypic" OR "male issue" OR "male issues" OR "male favoring" OR "sex insensitive" OR "sex harass" OR "sex harassment" OR "sex bias" OR "sex bases" OR "sex biasing" OR "gender based violence" OR "sex biased" OR "sex stereotype" OR "sex stereotyping" OR "sex stereotypical" OR "sex stereotypes" OR "sex stereotypes" OR "sex stereotypic" OR "sex issue" OR "sex issues" OR "sex inequality" OR "sex inequalities" OR "gender discriminate" OR "gender discrimination" OR "gender discriminations" OR "gender insensitive" OR "gender harass" OR "gender harassment" OR "gender biases" OR "gender biased" OR "gender biasing" OR "gender bias" OR "gender budget" OR "gender budgets" OR "gender mainstream" OR "gender mainstreaming" OR "gender stereotype" OR "gender stereotyping" OR "gender stereotypical" OR "gender stereotypes" OR "gender stereotypes" OR "gender stereotypic" OR "gender issue" OR "gender issues" OR "gender inequitable" OR "gender inequality" OR "gender inequalities" OR "gender in-equality" OR "gender in-equalities" OR "gender unequal")) AND (("Health examiners" OR "medical examiners" OR "clinic examiners" OR "clinical examiners" OR "Health assistants" OR "hospital assistants" OR "medical assistants" OR "clinic assistants" OR "clinical assistants" OR "Health administratORs" OR "hospital administratORs" OR "medical administratORs" OR "clinic administratORs" OR "clinical administratORs" OR "Health supervisORs" OR "hospital supervisORs" OR "medical supervisORs" OR "clinic supervisORs" OR "clinical supervisors" OR "Health teams" OR "Health examiner" OR "medical examiner" OR "clinic examiner" OR "clinical examiner" OR "Health assistant" OR "hospital assistant" OR "medical assistant" OR "clinic assistant" OR "clinical assistant" OR "Health administratOR" OR "hospital administratOR" OR "medical administratOR" OR "clinic administratOR" OR "clinical administratOR" OR "Health supervisOR" OR "hospital supervisOR" OR "medical supervisOR" OR "clinic supervisOR" OR "clinical supervisor" OR "Health team" OR "hospital team" OR "medical team" OR "clinic team" OR "clinical team" OR "inpatient team" OR "outpatient team" OR "Health staff" OR "hospital staff" OR "medical staff" OR "clinic staff" OR "clinical staff" OR "inpatient staff" OR "outpatient staff" OR "Health wORkers" OR "hospital wORkers" OR "medical wORkers" OR "clinic wORkers" OR "clinical wORkers" OR "Health researchers" OR "hospital researchers" OR "medical researchers" OR "clinic researchers" OR "clinical researchers" OR "health librarians" OR "hospital librarians" OR "medical librarians" OR "health infORmationists" OR "health technicians" OR "hospital technicians" OR "medical technicians" OR "health managers" OR "hospital managers" OR "medical managers" OR "hospital housekeepers" OR "hospital janitORs" OR "health officers" OR "hospital officers" OR "medical officers" OR "clinical officers" OR "medical responders" OR "clinical responders" OR "hospital responders" OR "medical paramedics" OR "hospital paramedics" OR "clinical labORatORians" OR "health coORdinatORs" OR "medical coORdinatORs" OR "clinical coORdinatORs" OR "Health wORker" OR "hospital wORker" OR "medical wORker" OR "clinic wORker" OR "clinical wORker" OR "Health researcher" OR "hospital researcher" OR "medical researcher" OR "clinic researcher" OR "clinical researcher" OR "health librarian" OR "hospital librarian" OR "medical librarian" OR "health infORmationist" OR "health technician" OR "hospital technician" OR "medical technician" OR "health manager" OR "hospital manager" OR "medical manager" OR "hospital housekeeper" OR "hospital janitOR" OR "health officer" OR "hospital officer" OR "medical officer" OR

"clinical officer" OR "medical responder" OR "clinical responder" OR "health responder" OR "medical paramedic" OR "hospital paramedic" OR "clinical labORatORian" OR "health coORdinatOR" OR "medical coORdinatOR" OR "clinical coORdinatOR" OR "health navigator" OR "clinical navigators" OR "health attendants" OR "clinical attendants" OR "health scientists" OR "clinical scientists" OR "hospital accountants" OR "hospital registrars" OR "clinical navigator" OR "health attendant" OR "clinical attendant" OR "health scientist" OR "clinical scientist" OR "hospital accountant" OR "hospital registrar" OR "health manpower" OR "health-care manpower" OR "clinical provider" OR "health provider" OR "healthcare provider" OR "health care provider" OR "clinical providers" OR "health providers" OR "healthcare providers" OR "health care providers" OR "health man-power" OR "health wORkfORce" OR "healthcare wORkfORce" OR "health-care wORkfORce" OR "clinical wORkfORce" OR "health caregivers" OR "clinical caregivers" OR "health care-givers" OR "clinical care-givers" OR "epidemiologists" OR "emergency dispatchers" OR "911 dispatchers" OR "midwife" OR "midwives" OR "health caregiver" OR "clinical caregiver" OR "health care-giver" OR "clinical care-giver" OR epidemiologist OR epidemiologists OR "emergency dispatcher" OR "911 dispatcher" OR physician OR physicians OR doctOR OR doctORs OR allopath OR allopaths OR osteopath OR osteopaths OR homeopath OR homeopaths OR "Clinical specialists" OR "health specialists" OR "healthcare specialists" OR "health-care specialists" OR "medical specialists" OR "Clinical practitioners" OR "health practitioners" OR "healthcare practitioners" OR "health-care practitioners" OR "medical practitioners" OR "Clinical generalists" OR "health generalists" OR "medical generalists" OR "medical students" OR "dental students" OR "Clinical specialist" OR "health specialist" OR "healthcare specialist" OR "health-care specialist" OR "medical specialist" OR "Clinical practitioner" OR "health practitioner" OR "healthcare practitioner" OR "health-care practitioner" OR "medical practitioner" OR "Clinical generalist" OR "health generalist" OR "medical generalist" OR "medical student" OR "dental student" OR nurse OR nurses OR "medical intern" OR "medical interns" OR "clinic intern" OR "medical graduate" OR "medical graduates" OR "clinical graduates" OR "dental graduate" OR "dental gradates" OR "medical fellow" OR "medical fellows" OR "clinical fellow" OR "clinical fellows" OR "house-officer" OR "house-officers" OR Resident OR Residents OR ORderly OR ORderlies OR dietician OR dieticians OR dietition OR dietitians OR psychologist OR psychologists OR CounselOR OR counselORs OR therapist OR therapists OR pharmacist OR pharmacists OR dentist OR dentists OR hygienist OR hygienists OR phlebotomist OR phlebotomists OR chiropracters OR acupuncturist OR acupuncturists OR anesthesiologist OR anesthesiologists OR aenesthesiologist OR aenesthesiologists OR anesthetist OR anesthetists OR anaesthetist OR anaesthetists OR perfusionist OR perfusionists OR surgeon OR surgeons OR paediatrician OR paediatricians OR pediatrician OR pediatricians OR obstetrician OR obstetricians OR Gynaecologist OR Gynaecologists OR Gynecologist OR Gynecologists OR radiologist OR radiologists OR sonographer OR sonographers OR cardiologists OR cardiologist OR gastroenterologist OR gastroenterologists OR hepatologist OR endocrinologist OR diabetologist OR internist OR hospitalist OR intensivist OR rheumatologist OR immunologist OR dermatologist OR allergist OR otolaryngologist OR otORhinolaryngologist OR oncologist OR hematologist OR haematologist OR ORthopedist OR ORthopaedist OR physiatrist OR podiatrist OR pathologist OR diener OR cytologist OR cytogeneticist OR geneticist OR haematopathologist OR hematopathologist OR neurologist OR neurosurgeon OR ophthalmologist OR optician OR urologist OR nephrologist OR pulmonologist OR neonatologist OR psychiatrist OR physiotherapist OR dentist OR prosthodontist OR periodontist OR endodontist OR nutritionist OR optometrist OR geriatrician OR gerontologist OR otologist OR feldsher OR exodontist OR doula OR "labOR coach" OR "labor coaches" OR MD OR anatomist OR anatomists OR audiologist OR audiologists OR cORoner OR cORoners OR hepatologists OR endocrinologists OR diabetologists OR internists OR hospitalists OR intensivists OR rheumatologists OR immunologists OR dermatologists OR allergists OR otolaryngologists OR otORhinolaryngologists OR oncologists OR hematologists OR haematologists OR ORthopedists OR

ORthopaedists OR physiatrists OR podiatrists OR pathologists OR dieners OR cytologists OR cytogeneticists OR geneticists OR haematopathologists OR hematopathologists OR neurologists OR neurosurgeons OR ophthalmologists OR opticians OR urologists OR nephrologists OR pulmonologists OR neonatologists OR psychiatrists OR physiotherapists OR dentists OR prosthodontists OR periodontists OR endodontist OR nutritionists OR optometrists OR geriatricians OR gerontologists OR otologists OR feldshers OR exodontists OR doulas OR "medical receptionist" OR "medical receptionists" OR "clinic receptionist" OR "clinic receptionists" OR "Health personnel" OR "hospital personnel" OR "medical personnel" OR "clinic personnel" OR "clinical personnel" OR "inpatient personnel" OR "outpatient personnel")) AND (("wORk hazard" OR "work hazards" OR "wORker hazard" OR "worker hazards" OR "wORker exposure" OR "wORk exposure" OR "work exposures" OR "wORking exposure" OR "working exposures" OR "wORk health" OR "wORker health" OR "wORker safety" OR "wORk safety" OR "wORking safety" OR "wORking hygiene" OR "wORk hygiene" OR "wORker hygiene" OR "Employee exposure" OR "employee exposures" OR "employee health" OR "employee safety" OR "occupational hazard" OR "occupational hazards" OR "occupational exposure" OR "occupational exposures" OR "occupational health" OR "occupational safety" OR "occupational hygiene" OR "industrial hazard" OR "industrial hazards" OR "industrial exposure" OR "industrial exposures" OR "industrial health" OR "industrial safety" OR "industrial hygiene" OR "aged wORker" OR "aged workers" OR employability OR "wORk capacity" OR "wORking capacity" OR "work capacities" OR "work capacities" OR "wORk resumption" OR "wORk load" OR "wORker capacity" OR "wORk ability" OR "wORking ability" OR "wORker ability" OR "wORk schedules" OR "wORking schedules" OR "wORk tables" OR "wORking tables" OR "wORking times" OR "wORk times" OR "wORker times" OR "wORk timings" OR "wORk weeks" OR "wORking weeks" OR "wORker weeks" OR "wORk days" OR "wORking days" OR "wORker days" OR "wORking loads" OR wORkload OR wORkloads OR wORkdays OR wORkweeks OR "wORker capacities" OR "wORk abilities" OR "wORking abilities" OR "wORker abilities" OR "wORk schedules" OR "wORking schedules" OR "wORk tables" OR "wORking tables" OR "wORking times" OR "wORk times" OR "wORker times" OR "wORk timings" OR "wORk weeks" OR "wORking weeks" OR "wORker weeks" OR "wORk days" OR "wORking days" OR "wORker days" OR "wORking loads" OR wORkdays OR wORkweeks)) AND ((battlefield OR battlefields OR "battle field" OR "battle fields" OR "combat field" OR "combat fields" OR "combat environment" OR "combat environments" OR "combat settings" OR "combat setting" OR "combat zone" OR "battle zone" OR "combat zones" OR "battle zones" OR terrORism OR bioterrORism OR genocide OR genocides OR "ethnic cleansing" OR "ethnic cleansings" OR holocaust OR holocausts OR Gun OR Guns OR gunshot OR gunshots OR "gun-shot" OR "gun-shots" OR Rifle OR Rifles OR shotgun OR shotguns OR shotgunning OR shotgunned OR firearm OR firearms OR Weapon OR weapons OR weaponized OR weaponized OR weaponised OR weaponised OR weaponization OR weaponisation OR bomb OR bombs OR explosive OR explosives OR bullet OR bullets OR "military attack" OR "military attacks" OR tORture OR war OR wars OR warfare OR "operation new dawn" OR "operation desert stORm" OR "operation iraqi freedom" OR "Afghan Campaign 2001" OR "American revolution" OR "American revolutions" OR "French revolution" OR "French revolutions" OR "9-11-2001" OR "11-September 2001" OR "September attack" OR "September attacks" OR immigrant OR immigrants OR emigrant OR emigrants OR "war victim" OR "war survivOR" OR "armed conflict victim" OR "war victims" OR "war survivORs" OR "armed conflict victims" OR violence OR refugee OR refugees OR "humanitarian crisis" OR "humanitarian crises" OR "complex emergency" OR "complex emergencies" OR "displaced population" OR "displaced populations" OR "displaced group" OR "displaced groups" OR "displaced people" OR "displaced person" OR "displaced persons" OR "undocumented population" OR "undocumented populations" OR "transient population" OR "transient populations" OR "transient group" OR "transient groups" OR "fORced displacement" OR "forced displacements" OR migrant OR migrants OR postconflict OR "post-conflict" OR "post-conflicts" OR postwar OR "post-war" OR "conflict

affected" OR "conflict-affected" OR "war affected" OR "war-affected" OR immigration OR immigrations OR emigrant OR emigrants OR "fragile state" OR "fragile states"))) OR (("occupational health") AND ("health care workers" OR "medical students") AND ("sex differences" OR "sexual dimorphism" OR "women" OR "men" OR "sexual roles" OR "sexual discrimination") AND ("war" OR "biological warfare" OR "chemical warfare" OR "terrorism" OR "explosives" OR "refugees" OR "migration" OR "migrants"))

Database #5: Global Index Medicus – 1988 to September 2019

tw:((war OR conflict OR terrorism OR warfare) AND (“health worker” OR “medical worker” OR “health personnel” OR “medical personnel”) AND (male OR female OR women OR men OR man OR woman OR sex OR gender))

Database #6: Ovid MEDLINE(R) and Epub Ahead of Print, In-Process & Other Non-Indexed Citations and Daily - 1946 to September 2019

(exp "warfare and armed conflicts"/ or exp Terrorism/ or exp Weapons/ or exp Explosive Agents/ or Torture/) OR (Refugees/ or "Transients and Migrants"/ or exp Human Migration/) OR (battlefield? or ((conflict? or battle? or combat?) adj3 (field? or environment? or setting? or context? or zone?)) or conflict* or (fragile adj2 state*) or terrorism or bioterroris* or genocide? or (ethnic* adj (violence* or cleans*))) OR (violence* or holocaust? or Gun? or Rifle? or shotgun? or firearm? or weapon? or bomb? or explosive? or bullet* or (humanitarian adj2 cris?s) or (complex adj (emergenc* or (security adj environment*))) or refugee? or ((displaced or undocumented or un-documented or transient or vulnerable) adj2 (population? or group? or nation? or people or person*)) or (asylum adj seeker?)) OR ((forced adj displacement) or migrant* or postconflict* or post-conflict* or postwar* or post-war* or ((conflict or war) adj affected) or immigrant* or emigrant* or ((warfare or war? or ((forced or armed) adj conflict?)) adj2 (victim? or survivor?))) OR ((militar* adj attack?) or torture or Warfare? or war or wars or ((operation* adj ((new adj dawn?) or ((Iraq* or endur*) adj freedom) or (desert adj (shield or storm)))) or (afghan* adj campaign adj "2001") or ((French or American) adj2 revolution*) or (9-11-2001 adj attack?) or ((September or Sep) adj3 attack?))).mp. AND (Sexism/ or gender based violence/ or Sex Characteristics/ or Men/ or Males/ or females/ or women/ or Sex Factors/ or exp Gender Identity/) OR ((sexism or (discriminat* or injust* or intoleran* or insensitiv* or harass* or bias* or favo?riti?m? or budget* or mainstream* or stereotyp* or issue or favoring or unfairness* or in-equit* or inequit* or unequal or un-equal)) adj4 (wom?n or m?n or female? or male? or gender? or sex)) OR ((gender adj based adj violence) or (sex* adj2 factor*) or (sexual adj dimorphism*) or masculin* or feminin* or ((characteristic* or differen* or d?morphism? or similarit* or compar* or outcome* or exposure* or perspective* or perception* or perceive* or sensitive* or dynamic* or risk factor* or stratification* or segregation* or conflict* or variab* or diversit* or relation* or variation* or approach* or disparit* or analys?s* or role*) adj3 (gender? or sex or (male? or m?n or (female? or wom?n))))).mp. AND (exp Health Personnel/ or exp Students, Health Occupations/ or health workforce/) OR (physician* or doctor* or allopath? or osteopath? or homeopath? or ((clinical* or health* or medical) adj (practitioner? or specialist? or generalist?)) or ((medical or nurs* or dental) adj (student? or graduate? or intern? or fellow?)) or (house adj officer?) or Resident? or nurse? or nursing or orderly or orderlies or dieti?ian* or psychologist* or counselor* or therapist* or pharmacist* or dentist* or hygeinist* or phlebotomist* or chiropracter* or acupuncturist* or an?esthesiologist* or an?esthetist* or perfusionist* or surgeon* or p?ediatrician* or obstetrician* or gyn?ecologist* or radiologist* or sonographer* or cardiologist* or gastroenterologist* or hepatologist* or endocrinologist* or diabetologist* or internist* or hospitalist* or intensivist* or rheumatologist* or immunologist* or dermatologist* or allergist* or otolaryngologist* or otorhinolaryngologist* or oncologist* or

h?ematologist* or orthop?edist* or physiatrist* or podiatrist* or pathologist* or diener* or cytologist* or cytogeneticist* or geneticist* or h?ematopathologist* or neurologist* or neurosurgeon* or ophthalmologist* or optician* or urologist* or nephrologist* or pulmonologist* or neonatologist* or psychiatrist* or physiotherapist* or dentist* prosthodontist* or periodontist* or endodontist or nutritionist* or optometrist* or geriatrician* or gerontologist* or otologist* or feldsher* or exodontist* or doula* or (labor adj3 coach*) or MD or anatomist* or audiologist* or coroner?) OR (((health* or hospital or medical or clinic* or inpatient* or outpatient*) adj3 (examiner* or assistant? or administrator* or supervisor* or receptionist* or personnel or team or staff or worker* or researcher or researchers or librarian* or informationist* or technician* or manager* or housekeeper* or janitor* or officer* or responder* or paramedic* or laboratorian* or coordinator* or navigator* or attendant* or scientist* or cashier? or accountant? or registrar? or provider* or chaperone? or man-power or manpower or workforce* or (care adj giver*) or caregiver?)) or epidemiologist* or ((ER or emergency or "911") adj dispatcher*) or midwi?e?).mp. AND (Occupational Health/) OR ((worker? or employee? or occupational or industrial) adj3 (hazard* or exposure* or health or safety or hygiene)) OR ((aged adj2 worker*) or employabilit* or (work* adj2 (capacit* or abilit* or resumption* or schedule* or table* or time* or timing? or week* or day? or load)) or workload or work-load or workday? or work-day? or workweek? or work-week*) OR 'quality of working life'.mp.

Database #7: PubMed – 1781 to September 2019

("warfare and armed conflicts"[Mesh] OR bioterrORism[Mesh:noexp] OR TerrORism[Mesh] OR Weapons[Mesh:noexp] OR Explosive Agents[Mesh] OR TORture[Mesh:noexp] OR (battlefield[tw] OR battlefields[tw] OR battle field*[tw] OR combat field*[tw] OR combat environment*[tw] OR combat setting*[tw] OR combat zone*[tw] OR battle zone[tw] OR terrORism[tw] OR bioterrORism[tw] OR genocide*[tw] OR ethnic cleansing*[tw] OR holocaust*[tw] OR Gun[tw] OR Guns[tw] OR gunshot[tw] OR gunshots[tw] OR gun-shot[tw] OR gun-shots[tw] OR Rifle[tw] OR Rifles[tw] OR shotgun[tw] OR shotguns[tw] OR shotgunning[tw] OR shotgunned[tw] OR firearm[tw] OR firearms[tw] OR Weapon[tw] OR weapons[tw] OR weaponised[tw] OR weaponized[tw] OR weaponization[tw] OR weaponisation[tw] OR bomb[tw] OR bombs[tw] OR explosive[tw] OR explosives[tw] OR bullet[tw] OR bullets[tw] OR military attack*[tw] OR tORture[tw] OR war[tw] OR wars[tw] OR warfare[tw] OR operation new dawn[tw] OR operation desert stORm[tw] OR operation iraqi freedom[tw] OR Afghan Campaign 2001-[tw] OR American revolution*[tw] OR French revolution*[tw] OR 9-11-2001 [tw] OR 11-September 2001[tw] OR September attack*[tw] OR Refugees[Mesh:noexp] OR "Transients and Migrants"[Mesh:noexp] OR Human Migration[Mesh] OR immigrant[tw] OR immigrants[tw] OR emigrant[tw] OR emigrants[tw] OR war victim*[tw] OR war survivOR*[tw] OR armed conflict victim*[tw] OR violence[tw] OR refugee[tw] OR refugees[tw] OR humanitarian crisis[tw] OR humanitarian crises[tw] OR complex emergenc*[tw] OR displaced population*[tw] OR displaced group*[tw] OR displaced people[tw] OR displaced person*[tw] OR undocumented population*[tw] OR transient population*[tw] OR transient group*[tw] OR fORced displacement*[tw] OR migrant[tw] OR migrants OR postconflict[tw] OR post-conflict*[tw] OR postwar[tw] OR post-war[tw] OR conflict affected[tw] OR conflict-affected[tw] OR war affected[tw] OR war-affected[tw] OR immigration[tw] OR immigrations[tw] OR emigrant[tw] OR emigrants[tw] OR fragile state*[tw]) AND ((Gender based violence[Mesh:noexp] OR (Gender Identity[Mesh] OR Sex FactORs[Mesh:noexp] OR Men[Mesh:noexp] OR Male[Mesh:noexp] OR Women[Mesh:noexp] OR Female[Mesh:noexp] OR Sexism[Mesh:noexp] OR Sex Characteristics[Mesh:noexp]) OR (gender-based violence[tw] OR Sex factOR*[tw] OR sexual dimORphism[tw] OR sexual dimORphisms[tw] OR masculin[tw] OR masculinity[tw] OR feminine[tw] OR feminin[tw] OR femininism[tw] OR femininity[tw] OR gender characteristic*[tw] OR gender difference*[tw] OR gender

similarit*[tw] OR gender comparison*[tw] OR gender outcome*[tw] OR gender exposure*[tw] OR gender perspective*[tw] OR gender perception*[tw] OR sex characteristic*[tw] OR sex difference*[tw] OR sex similarit*[tw] OR sex comparison*[tw] OR sex outcome*[tw] OR sex exposure[tw] OR sex perspective*[tw] OR sex perception*[tw] OR men difference*[tw] OR men similarit*[tw] OR men exposure[tw] OR man comparison[tw] OR man exposure*[tw] OR OR male characteristic*[tw] OR male difference*[tw] OR male comparison*[tw] OR male exposure*[tw] OR male perspective*[tw] OR male perception*[tw] OR female characteristic*[tw] OR female difference*[tw] OR female comparison*[tw] OR female outcome*[tw] OR female exposure*[tw] OR female perspective*[tw] OR female perception*[tw] OR women characteristic*[tw] OR women difference*[tw] OR women comparison*[tw] OR women outcome*[tw] OR women exposure*[tw] OR women perspective*[tw] OR women perception*[tw] OR woman characteristic*[tw] OR woman difference*[tw] OR woman similarit*[tw] OR woman comparison*[tw] OR woman outcome*[tw] OR woman perception*[tw] OR Gender sensitive[tw] OR gender dynamic*[tw] OR gender risk factOR*[tw] OR gender stratification[tw] OR gender segregation*[tw] OR gender conflict*[tw] OR gender variable*[tw] OR gender diversity[tw] OR gender diverse[tw] OR gender relation[tw] OR gender relations[tw] OR gender variation*[tw] OR gender approach[tw] OR gender disparit*[tw] OR gender analysis[tw] OR gender analyses[tw] OR gender role*[tw] OR sex sensitive[tw] OR sex dynamic*[tw] OR sex risk factOR*[tw] OR sex stratification[tw] OR sex segregation[tw] OR sex variable*[tw] OR sex diversity[tw] OR sex relation[tw] OR sex relations[tw] OR sex variation*[tw] OR sex disparit*[tw] OR sex analysis[tw] OR sex analyses[tw] OR sex role*[tw] OR male sensitive*[tw] OR male risk factOR*[tw] OR male diversity[tw] OR male relation[tw] OR male relations[tw] OR male variation[tw] OR male approach*[tw] OR male role*[tw] OR men sensitive*[tw] OR man sensitive*[tw] OR man role*[tw] OR female sensitive*[tw] OR female risk factOR*[tw] OR female variable*[tw] OR female relation[tw] OR female relations[tw] OR female variation*[tw] OR female approach[tw] OR female analysis[tw] OR female analyses[tw] OR female role*[tw] OR women sensitive*[tw] OR women analysis[tw] OR women analyses[tw] OR woman sensitive*[tw] OR woman role*[tw] OR sexism[tw] OR female harass*[tw] OR female stereotyp*[tw] OR female issue*[tw] OR female favORing*[tw] OR male discriminat*[tw] OR male harass*[tw] OR “male bias” OR “male biases” OR “male biased” OR “male biasing” OR male stereotyp*[tw] OR male issue*[tw] OR male favORing[tw] OR sex insensitive*[tw] OR sex harass*[tw] OR sex bias*[tw] OR sex stereotyp*[tw] OR sex issue*[tw] OR sex inequalit*[tw] OR gender discriminat*[tw] OR gender insensitive*[tw] OR gender harass*[tw] OR gender bias*[tw] OR gender budget*[tw] OR gender mainstream*[tw] OR gender stereotyp*[tw] OR gender issue*[tw] OR gender inequitable[tw] OR gender inequalit*[tw] OR gender in-equalit*[tw] OR gender unequal[tw])) AND ((Health Personnel[Mesh] OR Students, Health Occupations[Mesh] OR health wORkfORce[Mesh:noexp]) OR (Health examiner*[tw] OR medical examiner*[tw] OR clinic examiner*[tw] OR clinical examiner*[tw] OR Health assistant*[tw] OR hospital assistant*[tw] OR medical assistant*[tw] OR clinic assistant*[tw] OR clinical assistant*[tw] OR Health administratOR*[tw] OR hospital administratOR*[tw] OR medical administratOR*[tw] OR clinic administratOR*[tw] OR clinical administratOR*[tw] OR Health supervisOR*[tw] OR hospital supervisOR*[tw] OR medical supervisOR*[tw] OR clinic supervisOR*[tw] OR clinical supervisOR*[tw] OR Health team*[tw] OR hospital team[tw] OR medical team[tw] OR clinic team[tw] OR clinical team[tw] OR inpatient team[tw] OR outpatient team[tw] OR Health staff[tw] OR hospital staff[tw] OR medical staff[tw] OR clinic staff[tw] OR clinical staff[tw] OR inpatient staff[tw] OR outpatient staff[tw] OR Health wORker*[tw] OR hospital wORker*[tw] OR medical wORker*[tw] OR clinic wORker*[tw] OR clinical wORker*[tw] OR Health researcher*[tw] OR hospital researcher*[tw] OR medical researcher*[tw] OR clinic researcher*[tw] OR clinical researcher*[tw] OR health librarians[tw] OR health librarian[tw] OR hospital librarians[tw] OR hospital librarian[tw] OR medical librarians[tw] OR medical librarian[tw] OR health infORmationist*[tw] OR health

technician*[tw] OR hospital technician*[tw] OR medical technician*[tw] OR health manager*[tw] OR hospital manager*[tw] OR medical manager*[tw] OR hospital housekeeper*[tw] OR hospital janitOR*[tw] OR health officer*[tw] OR hospital officer*[tw] OR medical officer*[tw] OR clinical officer*[tw] OR medical responder*[tw] OR clinical responder*[tw] OR health responder*[tw] OR medical paramedic*[tw] OR hospital paramedic*[tw] OR clinical labORatORian*[tw] OR health coORdinatOR*[tw] OR medical coORdinatOR*[tw] OR clinical coORdinatOR*[tw] OR health navigatOR[tw] OR clinical navigatOR*[tw] OR health attendant*[tw] OR clinical attendant*[tw] OR health scientist*[tw] OR clinical scientist*[tw] OR hospital accountant*[tw] OR hospital registrar*[tw] OR health manpower[tw] OR health-care manpower[tw] OR clinical provider[tw] OR health provider[tw] OR healthcare provider[tw] OR health care provider[tw] OR clinical providers[tw] OR health providers[tw] OR healthcare providers[tw] OR health care providers[tw] OR health man-power[tw] OR health wORkfORce[tw] OR healthcare wORkfORce[tw] OR health-care wORkfORce[tw] OR clinical wORkfORce[tw] OR health caregiver*[tw] OR clinical caregiver*[tw] OR health care-giver*[tw] OR clinical care-giver*[tw] OR epidemiologist*[tw] OR epidemiologists[tw] OR emergency dispatcher*[tw] OR 911 dispatcher*[tw] OR midwife[tw] OR midwives[tw] OR physician[tw] OR physicians[tw] OR doctOR [tw] OR doctORs[tw] OR allopath[tw] OR allopaths[tw] OR osteopath[tw] OR osteopaths[tw] OR homeopath[tw] OR homeopaths[tw] OR Clinical specialist*[tw] OR health specialist*[tw] OR healthcare specialist*[tw] OR health-care specialist*[tw] OR medical specialist*[tw] OR Clinical practitioner*[tw] OR health practitioner*[tw] OR healthcare practitioner*[tw] OR health-care practitioner*[tw] OR medical practitioner*[tw] OR Clinical generalist*[tw] OR health generalist*[tw] OR medical generalist*[tw] OR medical student[tw] OR medical students[tw] OR dental student*[tw] OR nurse[tw] OR nurses[tw] OR medical intern[tw] OR medical interns[tw] OR clinic intern[tw] OR medical graduate[tw] OR medical graduates[tw] OR clinical graduates[tw] OR dental graduate*[tw] OR medical fellow[tw] OR medical fellows[tw] OR clinical fellow[tw] OR clinical fellows[tw] OR house-officer[tw] OR house-officers[tw] OR Resident[tw] OR Residents[tw] OR ORderly[tw] OR ORderlies[tw] OR dietician[tw] OR dieticians[tw] OR dietition[tw] OR dietitians[tw] OR psychologist[tw] OR psychologists[tw] OR CounselOR[tw] OR counselORs[tw] OR therapist[tw] OR therapists[tw] OR pharmacist[tw] OR pharmacists[tw] OR dentist[tw] OR dentists[tw] OR hygienist[tw] OR hygienists[tw] OR phlebotomist[tw] OR phlebotomists[tw] OR chiropracters[tw] OR acupuncturist[tw] OR acupuncturists[tw] OR anesthesiologist[tw] OR anesthesiologists[tw] OR aenesthesiologist[tw] OR aenesthesiologists[tw] OR anesthetist[tw] OR anesthetists[tw] OR anaesthetist[tw] OR anaesthetists[tw] OR perfusionist[tw] OR perfusionists[tw] OR surgeon[tw] OR surgeons[tw] OR paediatrician[tw] OR paediatricians[tw] OR pediatrician[tw] OR pediatricians[tw] OR obstetrician[tw] OR obstetricians[tw] OR Gynaecologist[tw] OR Gynaecologists[tw] OR Gynecologist[tw] OR Gynecologists[tw] OR radiologist[tw] OR radiologists[tw] OR sonographer[tw] OR sonographers[tw] OR cardiologists[tw] OR cardiologist[tw] OR gastroenterologist[tw] OR gastroenterologists[tw] OR hepatologist[tw] OR endocrinologist[tw] OR diabetologist[tw] OR internist[tw] OR hospitalist[tw] OR intensivist[tw] OR rheumatologist[tw] OR immunologist[tw] OR dermatologist[tw] OR allergist[tw] OR otolaryngologist[tw] OR otORhinolaryngologist[tw] OR oncologist[tw] OR hematologist[tw] OR haematologist[tw] OR ORthopedist[tw] OR ORthopaedist[tw] OR physiatrist[tw] OR podiatrist[tw] OR pathologist[tw] OR diener[tw] OR cytologist[tw] OR cytogeneticist[tw] OR geneticist[tw] OR haematopathologist[tw] OR hematopathologist[tw] OR neurologist[tw] OR neurosurgeon[tw] OR ophthalmologist[tw] OR optician[tw] OR urologist[tw] OR nephrologist[tw] OR pulmonologist[tw] OR neonatologist[tw] OR psychiatrist[tw] OR physiotherapist[tw] OR prosthodontist[tw] OR periodontist[tw] OR Endodontist[tw] OR nutritionist[tw] OR optometrist[tw] OR geriatrician[tw] OR gerontologist[tw] OR otologist[tw] OR feldsher[tw] OR exodontist[tw] OR doula[tw] OR labOR coach[tw] OR labor coaches[tw] OR MD[tw] OR anatomist[tw] OR anatomists[tw] OR audiologist[tw] OR audiologists[tw] OR cORoner[tw] OR

cORoners[tw] OR hepatologists[tw] OR endocrinologists[tw] OR diabetologists[tw] OR internists[tw] OR hospitalists[tw] OR intensivists[tw] OR rheumatologists[tw] OR immunologists[tw] OR dermatologists[tw] OR allergists[tw] OR otolaryngologists[tw] OR otORhinolaryngologists[tw] OR oncologists[tw] OR hepatologists[tw] OR endocrinologists[tw] OR diabetologists[tw] OR internists[tw] OR hospitalists[tw] OR intensivists[tw] OR rheumatologists[tw] OR immunologists[tw] OR dermatologists[tw] OR allergists[tw] OR otolaryngologists[tw] OR otORhinolaryngologists[tw] OR oncologists[tw] OR hematologists[tw] OR haematologists[tw] OR ORthopedists[tw] OR ORthopaedists[tw] OR physiatrists[tw] OR podiatrists[tw] OR pathologists[tw] OR dieners[tw] OR cytologists[tw] OR cytogeneticists[tw] OR geneticists[tw] OR haematopathologists[tw] OR hematopathologists[tw] OR neurologists[tw] OR neurosurgeons[tw] OR ophthalmologists[tw] OR opticians[tw] OR urologists[tw] OR nephrologists[tw] OR pulmonologists[tw] OR neonatologists[tw] OR psychiatrists[tw] OR physiotherapists[tw] OR prosthodontists[tw] OR periodontists[tw] OR endodontist OR nutritionists[tw] OR optometrists[tw] OR geriatricians[tw] OR gerontologists[tw] OR otologists[tw] OR feldshers[tw] OR exodontists[tw] OR doulas[tw] OR medical receptionist*[tw] OR clinic receptionist*[tw] OR Health personnel[tw] OR hospital personnel*[tw] OR medical personnel*[tw] OR clinic personnel*[tw] OR clinical personnel*[tw] OR inpatient personnel*[tw] OR outpatient personnel*[tw])) AND ((Occupational Health[Mesh:noexp]) OR (wORk hazard*[tw] OR wORker hazard*[tw] OR wORker exposure*[tw] OR wORk exposure*[tw] OR wORking exposure*[tw] OR wORk health[tw] OR wORker health[tw] OR wORker safety[tw] OR wORk safety[tw] OR wORking safety[tw] OR wORking hygiene[tw] OR wORk hygiene[tw] OR wORker hygiene[tw] OR Employee exposure*[tw] OR employee health[tw] OR employee safety[tw] OR occupational hazard*[tw] OR occupational exposure*[tw] OR occupational health[tw] OR occupational safety[tw] OR occupational hygiene[tw] OR industrial hazard*[tw] OR industrial exposure*[tw] OR industrial health[tw] OR industrial safety[tw] OR industrial hygiene[tw] OR aged wORker*[tw] OR employabilit*[tw] OR wORk capacit*[tw] OR wORking capacit*[tw] OR wORker capacit*[tw] OR wORk ability*[tw] OR wORking abilit*[tw] OR wORker ability*[tw] OR wORk resumption[tw] OR wORk schedule*[tw] OR wORking schedule*[tw] OR wORk table*[tw] OR wORking table*[tw] OR wORking time*[tw] OR wORk time*[tw] OR wORker time*[tw] OR wORk timing*[tw] OR wORk week*[tw] OR wORking week*[tw] OR wORker week*[tw] OR wORk day*[tw] OR wORking day*[tw] OR wORker day*[tw] OR wORk load[tw] OR wORking load*[tw] OR wORkload[tw] OR wORkloads[tw] OR wORkday*[tw] OR wORkweek*[tw]))

Database #8: Scopus –1788 to September 2019

(TITLE-ABS-KEY ("quality of working life" OR (work* W/2 (capacit* OR abilit* OR resumption* OR schedule* OR table* OR time* OR timing OR timings OR week* OR day OR days OR load)) OR (aged W/2 worker*) OR ((worker OR workers OR employee OR employees OR occupational OR industrial) W/3 (hazard* OR exposure* OR health OR safety OR hygiene)))) AND (TITLE-ABS-KEY (battlefield* OR ((conflict* OR battle* OR combat*) W/3 (field* OR environment OR environments OR setting* OR context OR contexts OR zone*)) OR conflict* OR (fragile W/2 state*) OR terrorism OR bioterroris* OR genocide* OR (ethnic* PRE/0 (violence* OR cleans*)) OR violence* OR holocaust* OR gun* OR rifle* OR shotgun* OR firearm* OR weapon* OR bomb* OR explosive* OR bullet* OR (humanitarian W/2 cris*s) OR ((displaced OR undocument* OR transient OR vulnerable) W/2 (population* OR group* OR nation* OR people OR person*)) OR migrant* OR postconflict* OR post-conflict* OR postwar* OR post-war* OR conflict-affected OR war-affected OR immigrant* OR emigrant* OR warfare OR war* OR (armed PRE/0 conflict*) OR (militar* PRE/0 attack*) OR torture OR ((iraq* OR endur*) PRE/0 freedom) OR (desert PRE/0 (shield OR storm)) OR (afghan* PRE/0 campaign PRE/0 2001) OR ((french OR american) W/2 revolution*) OR (9 11 2001 PRE/0 attack*) OR ((

september OR sep) W/3 attack*) OR (complex PRE/0 emergenc*) OR (complex PRE/0 security PRE/0 environment*) OR refugee* OR (asylum PRE/0 seeker*) OR (forced PRE/0 displacement))) AND ((TITLE-ABS-KEY ((sexism OR "gender based violence" OR (characteristic* OR differen* OR dimorphism* OR dymorphism* OR similarit* OR compar* OR outcome* OR exposure* OR perspective* OR perception* OR perceive* OR sensitive* OR dynamic* OR stratification* OR segregation* OR conflict OR variabl* OR diversit* OR relation* OR variation* OR approach* OR disparit* OR analysis OR analyses OR role*) W/3 (gender* OR sex OR male OR males OR m?n OR female OR females OR wom?n)) OR ((discriminat* OR injust* OR intoleran* OR insensitiv* OR harass* OR bias* OR favo*riti?m* OR budget* OR mainstream* OR stereotyp* OR issue OR favoring OR unfairness* OR in-equit* OR inequit* OR un-equal OR unequal) W/4 (wom?n OR m?n OR female* OR male* OR gender* OR sex)) OR (sex* W/2 factor*) OR masculin* OR feminin* OR (gender PRE/0 risk PRE/0 factor*) OR (sex PRE/0 risk PRE/0 factor*) OR (m?n PRE/0 risk PRE/0 factor*) OR (male* PRE/0 risk PRE/0 factor*) OR (female* PRE/0 risk PRE/0 factor*) OR (wom?n PRE/0 risk PRE/0 factor*) OR (sexual PRE/0 dimorphism*)))) AND (TITLE-ABS-KEY ((((medical OR nurs* OR dental) PRE/0 (student* OR graduate* OR intern OR interns OR fellow OR fellows)) OR (labor W/3 coach*) OR md OR anatomist* OR audiologist* OR coroner* OR ((health* OR hospital OR medical OR clinic* OR inpatient* OR outpatient*) W/3 (examiner* OR assistant* OR administrator* OR supervisor* OR receptionist* OR personnel OR team OR staff OR worker* OR researcher OR researchers OR librarian* OR informationist* OR technician* OR manager* OR housekeeper* OR janitor* OR officer* OR responder* OR paramedic* OR laboratorian* OR coordinator* OR navigator* OR attendant* OR scientist* OR cashier* OR accountant* OR registrar OR registrars OR provider* OR chaperone OR chaperones OR man-power OR manpower OR workforce* OR caregiver*)) OR ((er OR emergency OR "911") PRE/0 dispatcher*) OR epidemiologist* OR midwife* OR midwive* OR (medical PRE/0 care PRE/0 giver*) OR (hospital PRE/0 care PRE/0 giver*) OR (clinical PRE/0 care PRE/0 giver*) OR (house PRE/0 officer*) OR resident OR residents OR nurse OR nurses OR nursing OR orderly OR orderlies OR dieti*ian* OR psychologist* OR counselor* OR therapist* OR pharmacist* OR hygeinist* OR phlebotomist* OR chiropracter* OR acupuncturist* OR an*esthesiologist* OR an*esthetist* OR perfusionist* OR surgeon* OR p*ediatrician* OR obstetrician* OR gyn*ecologist* OR radiologist* OR sonographer* OR cardiologist* OR gastroenterologist* OR hepatologist* OR endocrinologist* OR diabetologist* OR internist* OR hospitalist* OR intensivist* OR rheumatologist* OR immunologist* OR dermatologist* OR allergist* OR otolaryngologist* OR otorhinolaryngologist* OR oncologist* OR h*ematologist* OR orthop*edist* OR physiatrist* OR podiatrist* OR pathologist* OR diener* OR cytologist* OR cytogeneticist* OR geneticist* OR h*ematopathologist* OR neurologist* OR neurosurgeon* OR ophthalmologist* OR optician* OR urologist* OR nephrologist* OR pulmonologist* OR neonatologist* OR psychiatrist* OR physiotherapist* OR dentist* OR prosthodontist* OR periodontist* OR endodontist OR nutritionist* OR optometrist* OR geriatrician* OR gerontologist* OR otologist* OR feldsher* OR exodontist* OR doula*)))

Database #9: Web of science Core Collection – 1900 to September 2019

Science Citation Index Expanded --1900-present
Social Sciences Citation Index --1900-present
Arts & Humanities Citation Index --1975-present
Conference Proceedings Citation Index- Science --1990-present
Conference Proceedings Citation Index- Social Science & Humanities --1990-present
Emerging Sources Citation Index --2015-present

TS=(battlefield$ OR ((conflict$ OR battle$ OR combat$) NEAR/3 (field$ OR environment$ OR setting$ OR context$ OR zone$)) OR conflict* OR (fragile NEAR/2 state*) OR terrORism OR bioterrORis* OR genocide$ OR “ethnic violence” OR “ethnic cleansing” OR violence* OR holocaust$ OR Gun$ OR Rifle$ OR shotgun$ OR firearm$ OR weapon$ OR bomb$ OR explosive$ OR bullet* OR (humanitarian NEAR/2 cris$s) OR “complex emergency” OR “complex security environment” OR “complex security environments” OR refugee$ OR ((displaced OR undocumented OR un-documented OR transient OR vulnerable) NEAR/2 (population$ OR group$ OR nation$ OR people OR person*)) OR “asylum seeker” OR “asylum seekers” OR “forced displacement” OR “forced displacements” OR migrant* OR postconflict* OR post-conflict* OR postwar* OR post-war* OR “conflict-affected” OR “war-affected” OR “conflict affected” OR “war affected” OR immigrant* OR emigrant* OR “warfare victim” OR “war victim” OR “warfare survivor” OR “war survivor” OR “Forced conflict victim” OR “forced conflict survivor” OR “armed conflict victim” OR “armed conflict survivor” OR “military attack” OR “warfare victims” OR “war victims” OR “warfare survivors” OR “war survivors” OR “Forced conflict victims” OR “forced conflict survivors” OR “armed conflict victims” OR “armed conflict survivors” OR “military attacks” OR torture OR Warfare$ OR war OR wars OR “operation new dawn” OR “operation Iraq freedom” OR “operation endure freedom” OR “operation desert shield” OR “operation desert storm” OR “afghan campaign 2001” OR “French revolution” OR “American revolution” OR “9-11-2001 attack” OR “operations new dawn” OR “operations Iraq freedom” OR “operations endure freedom” OR “operations desert shield” OR “operations desert storm” OR “afghan campaigns 2001” OR “French revolutions” OR “American revolutions” OR “9 11 2001 attacks” OR “September attack” OR “September attacks” OR “Sep attack” OR “Sep attacks”) AND TS=((sexism OR discriminat* OR injust* OR intoleran* OR insensitiv* OR harass* OR bias* OR favoritism OR favo$riti?m OR budget* or mainstream* or stereotyp* OR issue OR favoring OR unfairness* or in-equit* or inequit* or unequal or un-equal) NEAR/4 (woman OR women OR man OR men OR females OR female OR male OR males OR gender OR genders OR sex)) OR (sex NEAR/2 factor$) OR “sexual dimorphism" OR “sexual dimorphisms” OR masculin* OR feminin* OR ((characteristics OR differen* OR dimorphism$ OR dymorphism$ OR similarit* OR compar* OR outcome* OR exposure* OR perspective* OR perception* OR perceive* OR sensitive* OR dynamic* OR (risk NEAR/0 factor$) OR stratification* OR segregation* OR conflict* OR variab* OR diversit* OR relation* OR variation* OR approach* OR disparit* OR analys$s* OR role*) NEAR/3 (gender$ OR sex OR male$ OR m$n OR female$ OR wom$n) OR “gender based violence” OR “gender-based violence”) AND TS=(physician* OR doctOR* OR allopath$ OR osteopath$ OR homeopath$ OR “clinical practitioner” OR “clinical practitioners” OR “clinical specialist” OR “clinical specialists” OR “clinical generalist” OR “clinical generalists” OR “health practitioner” OR “health practitioners“ OR “healthcare practitioner” OR “healthcare practitioner” OR “health-care practitioner” OR “health-care practitioner” OR “health care practitioner” OR “health care practitioner” OR “health specialist” OR “health specialists” OR “healthcare specialist” OR “healthcare specialists” OR “health-care specialist” OR “health-care specialists” OR “health care specialist” OR “health care specialists” OR “health generalist” OR “health generalists” OR “healthcare generalist” OR “healthcare generalists” OR “health-care generalist” OR “health-care generalists” OR “health-care generalist” OR “health-care generalists” OR “medical practitioner” OR “medical specialist” OR “medical generalist” OR “medical student” OR “medical graduate” OR “medical intern” OR “medical fellow” OR “medical practitioners” OR “medical specialists” OR “medical generalists” OR “medical students” OR “medical graduates” OR “medical interns” OR “medical fellows” OR “nurse student” OR “nurse students” OR “nursing student” OR “nursing students” OR “nurse graduate” OR “nurse graduates” OR “nursing graduate” OR “nursing graduates” OR “nurse intern” OR “nurse interns” OR “nursing intern” OR “nursing interns” OR “nurse fellow” OR “nurse fellows” OR “nursing fellow” OR “nursing fellows” OR “dental student” OR “dental students” OR “dental graduate” OR “dental intern” OR “dental fellow” OR “house officer” OR

“dental graduates” OR “dental interns” OR “dental fellows” OR “house officers” OR Resident$ OR nurse$ OR nursing OR ORderly OR ORderlies OR dieti$ian* OR psychologist* OR counselOR* OR therapist* OR pharmacist* OR dentist* OR hygeinist* OR phlebotomist* OR chiropracter* OR acupuncturist* OR an$esthesiologist* OR an$esthetist* OR perfusionist* OR surgeon* OR p$ediatrician* OR obstetrician* OR gyn$ecologist* OR radiologist* OR sonographer* OR cardiologist* OR gastroenterologist* OR hepatologist* OR endocrinologist* OR diabetologist* OR internist* OR hospitalist* OR intensivist* OR rheumatologist* OR immunologist* OR dermatologist* OR allergist* OR otolaryngologist* OR otORhinolaryngologist* OR oncologist* OR h$ematologist* OR ORthop$edist* OR physiatrist* OR podiatrist* OR pathologist* OR diener* OR cytologist* OR cytogeneticist* OR geneticist* OR h$ematopathologist* OR neurologist* OR neurosurgeon* OR ophthalmologist* OR optician* OR urologist* OR nephrologist* OR pulmonologist* OR neonatologist* OR psychiatrist* OR physiotherapist* OR prosthodontist* OR periodontist* OR endodontist OR nutritionist* OR optometrist* OR geriatrician* OR gerontologist* OR otologist* OR feldsher* OR exodontist* OR doula* OR (labOR NEAR/3 coach*) OR MD OR anatomist* OR audiologist* OR cORoner$ OR ((health* OR hospital OR medical OR clinic* OR inpatient* OR outpatient*) NEAR/3 (examiner* OR assistant$ OR administratOR* OR supervisOR* OR receptionist* OR personnel OR team OR staff OR wORker* OR researcher OR researchers OR librarian* OR infORmationist* OR technician* OR manager* OR housekeeper* OR janitOR* OR officer* OR responder* OR paramedic* OR labORatORian* OR coORdinatOR* OR navigatOR* OR attendant* OR scientist* OR cashier$ OR accountant$ OR registrar$ OR provider* OR chaperone$ OR man-power OR manpower OR wORkfORce* OR (care NEAR/0 giver$) OR caregiver$)) OR epidemiologist* OR “ER dispatcher” OR “ER dispatchers” OR “emergency dispatcher” OR “emergency dispatchers” OR “911 dispatcher” OR “911 dispatchers” OR midwi?e$) AND TS=(((worker$ OR employee$ OR occupational OR industrial) NEAR/3 (hazard* OR exposure* OR health OR safety OR hygiene)) OR (aged NEAR/2 worker*) OR employabilit* OR (work* NEAR/2 (capacit* OR abilit* OR resumption* OR schedule* OR table* OR time* OR timing$ OR week* OR day$ OR load)) OR workload* OR work-load* OR workday$ OR work-day$ OR workweek$ OR work-week* OR “'quality of working life”)

Supplementary File 2: List of Included Studies

1. Aldrich, T.K.; Gustave, J.; Hall, C.B.; Cohen, H.W.; Webber, M.P.; Zeig-Owens, R.; Cosenza, K.; Christodoulou, V.; Glass, L.; Al-Othman, F., et al. Lung function in rescue workers at the World Trade Center after 7 years. *New England Journal of Medicine* **2010**, *362*, 1263-1272.
2. Berrios-Torres, S.I.; Greenko, J.A.; Phillips, M.; Miller, J.R.; Treadwell, T.; Ikeda, R.M. World Trade Center rescue worker injury and illness surveillance, New York, 2001. *American Journal of Preventive Medicine* **2003**, *25*, 79-87.
3. Biggs, Q.M.; Fullerton, C.S.; Reeves, J.J.; Grieger, T.A.; Reissman, D.; Ursano, R.J. Acute Stress Disorder, Depression, and Tobacco Use in Disaster Workers Following 9/11. *Am J Orthopsychiat* **2010**, *80*, 586-592, doi:10.1111/j.1939-0025.2010.01063.x.
4. Burnham, G.; Malik, S.; Al-Shibli, A.S.; Mahjoub, A.R.; Baqer, A.Q.; Baqer, Z.Q.; Al Qaraghuli, F.; Doocy, S. Understanding the impact of conflict on health services in Iraq: information from 401 Iraqi refugee doctors in Jordan. *International Journal of Health Planning & Management* **2012**, *27*, e51-64.
5. Crowley, L.E.; Herbert, R.; Moline, J.M.; Wallenstein, S.; Shukla, G.; Schechter, C.; Skloot, G.S.; Udasin, I.; Luft, B.J.; Harrison, D., et al. "Sarcoid like" granulomatous pulmonary disease in World Trade Center disaster responders. *American Journal of Industrial Medicine* **2011**, *54*, 175-184, doi:10.1002/ajim.20924.Published.
6. Daly, E.S.; Gulliver, S.B.; Zimering, R.T.; Knight, J.; Kamholz, B.W.; Morissette, S.B. Disaster mental health workers responding to Ground Zero: one year later. *J Trauma Stress* **2008**, *21*, 227-230, doi:10.1002/jts.20311.
7. Devkota, B.; van Teijlingen, E. Demystifying the Maoist barefoot doctors of Nepal. *Med Confl Surviv* **2010**, *26*, 108-123, doi:10.1080/13623699.2010.491382.
8. Donaldson, R.I.; Shanovich, P.; Shetty, P.; Clark, E.; Aziz, S.; Morton, M.; Hasoon, T.; Evans, G. A survey of national physicians working in an active conflict zone: the challenges of emergency medical care in Iraq. *Prehosp Disaster Med* **2012**, *27*, 153-161, doi:10.1017/S1049023X12000519.
9. Footer, K.H.; Meyer, S.; Sherman, S.G.; Rubenstein, L. On the frontline of eastern Burma's chronic conflict--listening to the voices of local health workers. *Soc Sci Med* **2014**, *120*, 378-386, doi:10.1016/j.socscimed.2014.02.019.
10. Gibbons, S.W.; Hickling, E.J.; Barnett, S.D.; Herbig-Wall, P.L.; Watts, D.D. Gender differences in response to deployment among military healthcare providers in Afghanistan and Iraq. *Journal of women's health (2002)* **2012**, *21*, 496-504, doi:10.1089/jwh.2011.3097.
11. Glaser, M.S.; Shah, N.; Webber, M.P.; Zeig-Owens, R.; Jaber, N.; Appel, D.W.; Hall, C.B.; Weakley, J.; Cohen, H.W.; Shulman, L., et al. Obstructive sleep apnea and World Trade Center exposure. *J Occup Environ Med* **2014**, *56 Suppl 10*, S30-34, doi:10.1097/JOM.0000000000000283.
12. Grieger, T.A.; Kolkow, T.T.; Spira, J.L.; Morse, J.S.; Kolkow, T.T.; Spira, J.L.; Morse, J.S.; Grieger, T.A. Post-traumatic stress disorder and depression in health care providers returning from deployment to Iraq and Afghanistan. *Military Medicine* **2007**, *172*, 451-455.
13. Hamdan, M.; Abu Hamra, A. Workplace violence towards workers in the emergency departments of Palestinian hospitals: a cross-sectional study. *Human resources for health* **2015**, *13*, 28, doi:10.1186/s12960-015-0018-2.
14. Hamdan, M.; Hamra, A.A. Burnout among workers in emergency Departments in Palestinian hospitals: prevalence and associated factors. *BMC Health Services Research* **2017**, *17*, 407.

15. Kitaneh, M.; Hamdan, M. Workplace violence against physicians and nurses in Palestinian public hospitals: a cross-sectional study. *BMC health services research* **2012**, *12*, 469, doi:10.1186/1472-6963-12-469.
16. Lafta, R.K.; Falah, N. Violence against health-care workers in a conflict affected city. *Medicine, Conflict and Survival* **2019**, *35*, 65-79, doi:10.1080/13623699.2018.1540095.
17. Lewis, P.C.; Stewart, D.; Brown, W. Deployment experiences of Army nurse practitioners. *Military medicine* **2012**, *177*, 889-893, doi:10.7205/milmed-d-12-00047.
18. Macera, C.A.; Aralis, H.J.; Highfill-McRoy, R.; Rauh, M.J. Posttraumatic stress disorder after combat zone deployment among navy and marine corps men and women. *Journal of Women's Health* **2014**, *23*, 499-505, doi:10.1089/jwh.2013.4302.
19. Maes, K.; Kalofonos, I. Becoming and remaining community health workers: Perspectives from Ethiopia and Mozambique. *Social Science and Medicine* **2013**, *87*, 52-59, doi:10.1016/j.socscimed.2013.03.026.
20. Maupin, G.M.; Tvaryanas, A.P.; White, E.D.; Mahaney, H.J. Assessment of Deployment-Related Exposures on Risk of Incident Mental Health Diagnoses Among Air Force Medical Service Personnel: Nested Case-Control Study. *Military medicine* **2018**, *183*, e123-e132, doi:10.1093/milmed/usx056.
21. Michlig, G.J.; Lafta, R.; Al-Nuaimi, M.; Burnham, G. Providing healthcare under ISIS: A qualitative analysis of healthcare worker experiences in Mosul, Iraq between June 2014 and June 2017. *Global Public Health* **2019**, *14*, 1414-1427, doi:10.1080/17441692.2019.1609061.
22. Misra, M.; Greenberg, N.; Hutchinson, C.; Brain, A.; Glozier, N. Psychological impact upon London Ambulance Service of the 2005 bombings. *Occup Med (Lond)* **2009**, *59*, 428-433, doi:10.1093/occmed/kqp100.
23. Najafizada, S.A.; Labonte, R.; Bourgeault, I.L. Community health workers of Afghanistan: a qualitative study of a national program. *Confl Health* **2014**, *8*, 26, doi:10.1186/1752-1505-8-26.
24. Namakula, J.; Witter, S. Living through conflict and post-conflict: experiences of health workers in northern Uganda and lessons for people-centred health systems. *Health Policy Plan* **2014**, *29 Suppl 2*, ii6-14, doi:10.1093/heapol/czu022.
25. Namakula, J.; Witter, S.; Ssengooba, F. Health worker experiences of and movement between public and private not-for-profit sectors-findings from post-conflict Northern Uganda. *Human resources for health* **2016**, *14*, 1-11, doi:10.1186/s12960-016-0114-y.
26. Pedersen, M.J.B.; Gjerland, A.; Rund, B.R.; Ekeberg, Ø.; Skogstad, L. Emergency preparedness and role clarity among rescue workers during the terror attacks in Norway July 22, 2011. *PLoS ONE* **2016**, *11*, doi:10.1371/journal.pone.0156536.
27. Putman, B.; Zeig-Owens, R.; Singh, A.; Hall, C.B.; Schwartz, T.; Webber, M.P.; Cohen, H.W.; Prezant, D.J.; Bachert, C.; Weiden, M.D. Risk factors for post-9/11 chronic rhinosinusitis in Fire Department of the City of New York workers. *Occup Environ Med* **2018**, *75*, 884-889, doi:10.1136/oemed-2018-105297.
28. Raven, J.; Wurie, H.; Witter, S. Health workers' experiences of coping with the Ebola epidemic in Sierra Leone's health system: a qualitative study. *BMC Health Services Research* **2018**, *18*, 251.
29. Razik, S.; Ehring, T.; Emmelkamp, P.M. Psychological consequences of terrorist attacks: prevalence and predictors of mental health problems in Pakistani emergency responders. *Psychiatry Research* **2013**, *207*, 80-85.
30. Sargent, P.; Millegan, J.; Delaney, E.; Roesch, S.; Sanders, M.; Mak, H.; Mallahan, L.; Raducha, S.; Webb-Murphy, J. Health Care Provider Burnout in a United States Military Medical Center During a Period of War. *Military medicine* **2016**, *181*, 136-142, doi:10.7205/milmed-d-14-00449.

31. Slusarcick, A.L.; Ursano, R.J.; Fullerton, C.S.; Dinneen, M.P. Stress and coping in male and female health care providers during the Persian Gulf War: The USNS Comfort hospital ship. *Military Medicine* **1999**, *164*, 166-173.
32. Smith, E.C.; Burkle, F.M., Jr. Paramedic and emergency medical technician reflections on the ongoing impact of the 9/11 terrorist attacks. *Prehospital and Disaster Medicine* **2019**, *34*, 56-61, doi:10.1017/S1049023X18001255.
33. Sousa, C.; Hagopian, A. Conflict, health care and professional perseverance: a qualitative study in the West Bank. *Global Public Health* **2011**, *6*, 520-533.
34. Thomyangkoon, P.; Kongsakon, R.; Pornputkul, V.; Putthavarang, T. Quality of life, needs and the mode of coping of the health personnel at Naradhiwasrajanagarindra Hospital in terrorism influence area in Thailand. *Journal of the Medical Association of Thailand* **2012**, *95 Suppl 3*, S102-108.
35. Tvaryanas, A.P.; Maupin, G.M.; Fouts, B.L. Assessment of Deployment-Related Exposures on Risk of Incident Mental Health Diagnoses Among Air Force Critical Care Providers: Nested Case-Control Study. *Military medicine* **2016**, *181*, 143-151, doi:10.7205/milmed-d-14-00585.
36. Veronese, G. Self-perceptions of well-being in professional helpers and volunteers operating in war contexts. *Journal of Health Psychology* **2013**, *18*, 911-925, doi:10.1177/1359105312457804.
37. Vossbrinck, M.; Zeig-Owens, R.; Hall, C.B.; Schwartz, T.; Moir, W.; Webber, M.P.; Cohen, H.W.; Nolan, A.; Weiden, M.D.; Christodoulou, V., et al. Post-9/11/2001 lung function trajectories by sex and race in World Trade Center-exposed New York City emergency medical service workers. *Occupational and Environmental Medicine* **2017**, *74*, 200-203, doi:10.1136/oemed-2016-103619.
38. Wang, K.; Gimbel, S.; Malik, E.; Hassen, S.; Hagopian, A. The experience of Chinese physicians in the national health diplomacy programme deployed to Sudan. *Global Public Health* **2012**, *7*, 196-211.
39. Webber, M.P.; Glaser, M.S.; Weakley, J.; Soo, J.; Ye, F.; Zeig-Owens, R.; Weiden, M.D.; Nolan, A.; Aldrich, T.K.; Kelly, K., et al. Physician-diagnosed respiratory conditions and mental health symptoms 7-9 years following the World Trade Center disaster. *American Journal of Industrial Medicine* **2011**, *54*, 661-671.
40. Webber, M.P.; Moir, W.; Crowson, C.S.; Cohen, H.W.; Zeig-Owens, R.; Hall, C.B.; Berman, J.; Qayyum, B.; Jaber, N.; Matteson, E.L., et al. Post-September 11, 2001, Incidence of Systemic Autoimmune Diseases in World Trade Center-Exposed Firefighters and Emergency Medical Service Workers. *Mayo Clin Proc* **2016**, *91*, 23-32, doi:10.1016/j.mayocp.2015.09.019.
41. Wheeler, K.; McKelvey, W.; Thorpe, L.; Perrin, M.; Cone, J.; Kass, D.; Farfel, M.; Thomas, P.; Brackbill, R. Asthma diagnosed after 11 September 2001 among rescue and recovery workers: findings from the World Trade Center Health Registry. *Environmental Health Perspectives* **2007**, *115*, 1584-1590.
42. Witter, S.; Namakula, J.; Wurie, H.; Chirwa, Y.; So, S.; Vong, S.; Ros, B.; Buzuzi, S.; Theobald, S. The gendered health workforce: mixed methods analysis from four fragile and post-conflict contexts. *Health Policy and Planning* **2017**, *32*, V52-V62, doi:10.1093/heapol/czx102.
43. Witter, S.; Wurie, H.; Chandiwana, P.; Namakula, J.; So, S.; Alonso-Garbayo, A.; Ssengooba, F.; Raven, J. How do health workers experience and cope with shocks? Learning from four fragile and conflict-affected health systems in Uganda, Sierra Leone, Zimbabwe and Cambodia. *Health Policy Plan* **2017**, *32*, iii3-iii13, doi:10.1093/heapol/czx112.
44. Witter, S.; Wurie, H.; Namakula, J.; Mashange, W.; Chirwa, Y.; Alonso-Garbayo, A.; Alonso-Garbayo, A. Why do people become health workers? Analysis from life histories in 4 post-conflict and post-crisis countries. *International Journal of Health Planning & Management* **2018**, *33*, 449-459, doi:10.1002/hpm.2485.

45. Wurie, H.R.; Samai, M.; Witter, S. Retention of health workers in rural Sierra Leone: findings from life histories. *Human resources for health* **2016**, *14*, 3, doi:10.1186/s12960-016-0099-6.
46. Yip, J.; Zeig-Owens, R.; Hall, C.B.; Webber, M.P.; Olivieri, B.; Schwartz, T.; Kelly, K.J.; Prezant, D.J. Health Conditions as Mediators of the Association Between World Trade Center Exposure and Health-Related Quality of Life in Firefighters and EMS Workers. *J Occup Environ Med* **2016**, *58*, 200-206, doi:10.1097/JOM.0000000000000597.
47. Yip, J.; Zeig-Owens, R.; Webber, M.P.; Kablanian, A.; Hall, C.B.; Vossbrinck, M.; Xiaoxue, L.; Weakley, J.; Schwartz, T.; Kelly, K.J., et al. World Trade Center-related physical and mental health burden among New York City Fire Department emergency medical service workers. *Occupational & Environmental Medicine* **2016**, *73*, 13-20, doi:10.1136/oemed-2014-102601.
